# Advances in biomimetic mineralized biomaterials for bone tissue engineering

**DOI:** 10.1093/rb/rbag093

**Published:** 2026-05-12

**Authors:** Zefang Li, Wei Sun, Shuaicong Huang, Yaning Zhao, Xiaoyi Wu, Cui Huang, Hongye Yang

**Affiliations:** State Key Laboratory of Oral & Maxillofacial Reconstruction and Regeneration, Key Laboratory of Oral Biomedicine Ministry of Education, Hubei Key Laboratory of Stomatology, School & Hospital of Stomatology, Wuhan University, Wuhan 430079, China; State Key Laboratory of Oral & Maxillofacial Reconstruction and Regeneration, Key Laboratory of Oral Biomedicine Ministry of Education, Hubei Key Laboratory of Stomatology, School & Hospital of Stomatology, Wuhan University, Wuhan 430079, China; State Key Laboratory of Oral & Maxillofacial Reconstruction and Regeneration, Key Laboratory of Oral Biomedicine Ministry of Education, Hubei Key Laboratory of Stomatology, School & Hospital of Stomatology, Wuhan University, Wuhan 430079, China; State Key Laboratory of Oral & Maxillofacial Reconstruction and Regeneration, Key Laboratory of Oral Biomedicine Ministry of Education, Hubei Key Laboratory of Stomatology, School & Hospital of Stomatology, Wuhan University, Wuhan 430079, China; State Key Laboratory of Oral & Maxillofacial Reconstruction and Regeneration, Key Laboratory of Oral Biomedicine Ministry of Education, Hubei Key Laboratory of Stomatology, School & Hospital of Stomatology, Wuhan University, Wuhan 430079, China; State Key Laboratory of Oral & Maxillofacial Reconstruction and Regeneration, Key Laboratory of Oral Biomedicine Ministry of Education, Hubei Key Laboratory of Stomatology, School & Hospital of Stomatology, Wuhan University, Wuhan 430079, China; State Key Laboratory of Oral & Maxillofacial Reconstruction and Regeneration, Key Laboratory of Oral Biomedicine Ministry of Education, Hubei Key Laboratory of Stomatology, School & Hospital of Stomatology, Wuhan University, Wuhan 430079, China

**Keywords:** biomaterials, bone tissue engineering, bone regeneration, biomineralization, biomimetic mineralization

## Abstract

The intricate orchestration of bone biomineralization—where cells, signaling molecules and organic matrix tightly cooperate to produce native bone with remarkable strength and dynamic biological activities—exemplifies a pinnacle of evolutionary engineering. Inspired by this living blueprint, biomimetic mineralized biomaterials (BMBs) have emerged as a key strategy in bone tissue engineering, aiming not only to replicate the hybrid composition and multiscale architecture of native bone but also to emulate aspects of its formation and biological functions. This review first outlines the fundamental mechanisms of bone biomineralization as a conceptual basis for biomimicry. It then summarizes recent advances in material systems and fabrication strategies that enable increasingly precise control over minerals. We further discuss the roles of BMBs in shaping the regenerative microenvironment, including their regulation of osteogenesis, angiogenesis, immunomodulation and neuroregeneration. Despite notable progress, enduring challenges include achieving precise structural mimicry, faithfully reproducing the dynamic biomineralization and seamlessly integrating multiple functions within systems. Addressing these challenges is poised to bridge the gap from concept to clinic, guiding the development of biomimetic systems that operate in harmony with the body’s native microenvironment and realizing the promise of ‘learning from nature to restore life’.

## Graphical abstract

**Graphical Abstract:**
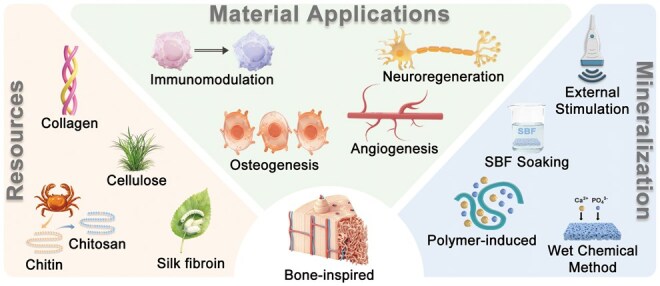

## Introduction

As the masterpiece of biomineralization, bone fulfills diverse and indispensable roles in the human body, ranging from providing mechanical support and facilitating physical movement to protecting vital organs and regulating hematopoiesis. However, these critical functions are often compromised by bone defects, which arise from traumas, diseases and accidental injuries. Studies have shown that minor bone defects (<5 mm) can undergo endogenous repair through the mobilization of various cells. Still, defects exceeding 50% of the cortical circumference [1], known as critical-size defects (CSDs), fail to heal spontaneously and therefore require therapeutic intervention [2]. Such significant defects can cause limb dysfunction, chronic pain and nonunion of fractures, which severely impair patients’ overall health and quality of life. The lack of skeletal protection also increases the susceptibility of internal organs to external forces. Globally, bone injuries affect approximately 150 million individuals each year [3], among which CSDs account for a considerable proportion [4, 5] and impose a substantial socioeconomic burden. Among existing clinical options, autologous bone grafting is limited by donor availability, whereas allogeneic/xenogeneic bone grafting carries risks of disease transmission and immune rejection.

To address these limitations, bone tissue engineering (BTE) has emerged as a promising therapeutic approach. In BTE, biomaterials serve as temporary scaffolds that support the adhesion, proliferation and differentiation of bone cells [6]. Ideally, these materials should not only fulfill fundamental requirements such as biocompatibility, osteoconductivity and osteoinductivity, but also actively orchestrate the bone healing cascade by providing coordinated support and regulatory cues throughout the regenerative process. Furthermore, mechanical and structural properties are also required to recapitulate native bone [7, 8]. Among fabrication strategies, biomimetic mineralization has attracted particular attention for its ability to emulate the natural properties of bone. Herein, we define biomimetic mineralized biomaterials (BMBs) as a class of bioactive materials engineered through biomimetic mineralization to recapitulate the chemical composition, hierarchical architecture, surface morphology, mechanical properties and even mineralization of native bone, while simultaneously interacting with living systems to provide biologically responsive and functionally adaptive cues for clinically relevant bone regeneration.

**Figure 1:**
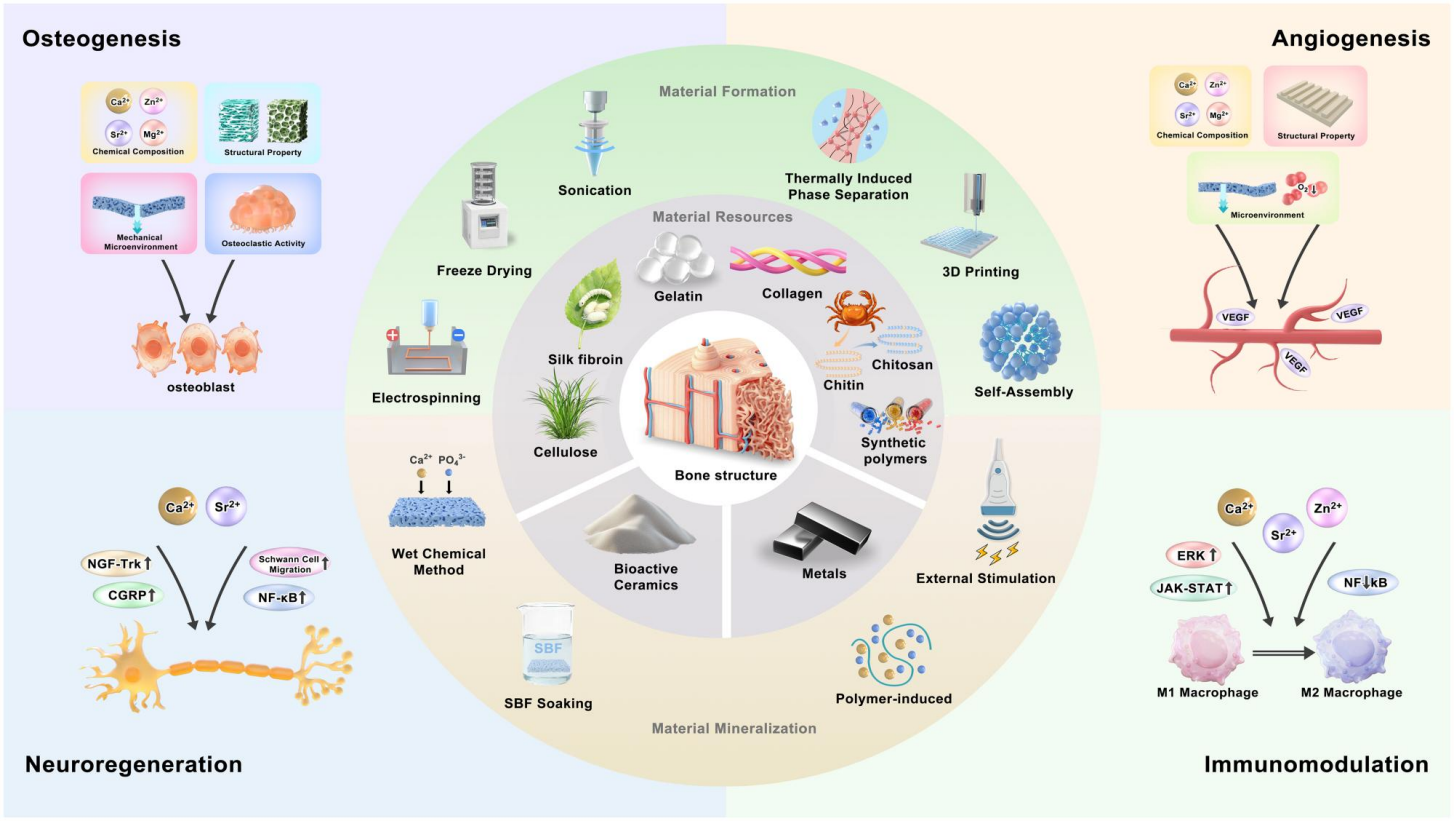
Inspired by physiological bone, BMBs feature a broad spectrum of source materials, fabrication strategies and biomedical applications.

Recent studies have demonstrated that BMBs not only enhance osteoblast proliferation and differentiation but also exhibit unique advantages beyond conventional biomaterials. Specifically, their ability to release bioactive ions and mimic the hierarchical architecture of bone enables them to modulate key biological processes in tissue regeneration [9]. This review provides a comprehensive overview of recent advances in BMBs for BTE, with a particular focus on their design principles and multifunctional biological roles. It begins by outlining the fundamental mechanisms of biomineralization and the key concepts underlying biomimetic material design. Building on this foundation, major classes of BMBs are systematically discussed, highlighting the relationship among material composition, structure and functional performance. The review then explores the various biological functions of BMBs, including their roles in osteogenesis, angiogenesis, immunomodulation and neuroregeneration, with a special focus on how these processes are coordinated and occur in sequence during bone healing. Finally, it discusses challenges and future directions for clinical application, including biosafety, scalability, regulation and long-term effectiveness. By integrating insights from materials science, biology and clinical research, this review emphasizes that BMBs possess multiple bioactivities that are beneficial for bone healing (Figure 1). Future materials should, therefore, be systematically evaluated for combined and sequential effects on key biological processes to ensure comprehensive validation of their preclinical assessment.

## Bone and biomineralization

Before exploring BMBs, it is necessary to first understand the structure, composition and biomineralization of bone.

### Bone structure and bone healing

Bone is an essential tissue that protects internal organs and provides mechanical support. It is also considered the largest and most important endocrine organ in the human body. Spongy bone consists of many interwoven needle- and plate-like trabeculae that form a porous structure, while the fundamental unit of compact bone is the Haversian system. Between the lamellae of this system are bone lacunae that house osteocytes and communicate through canaliculi. Despite structural differences, both types of bone share a common chemical foundation of mineralized collagen fibers. The bone matrix contains various noncollagenous proteins (NCPs) that regulate bone growth, with bone morphogenetic proteins (BMPs) being the most extensively studied. BMPs bind to serine/threonine kinase receptors on the cell membrane and thereby promote the differentiation of bone marrow mesenchymal stem cells (BMSCs) into osteoblasts [10].

**Figure 2:**
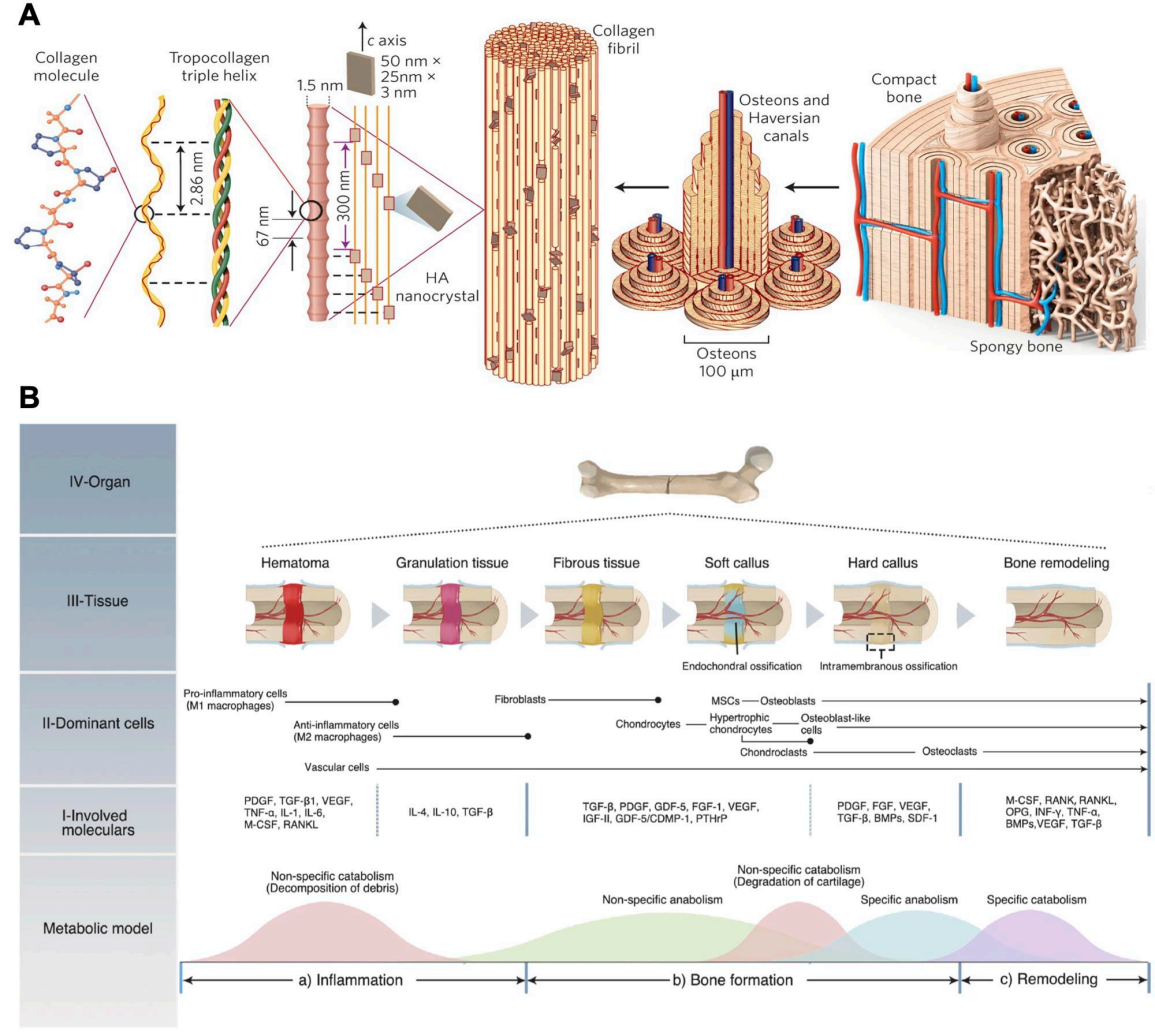
Bone structure and bone healing mechanisms. (**A**) The hierarchical architecture of bone, illustrating its chemical composition and structural features; reproduced with permission from Ref. [22], Copyright 2015, Springer Nature. (**B**) The stages of bone healing proceed sequentially and involve dynamic metabolic changes; reproduced with permission from Ref. [23], Copyright 2021, Elsevier.

The structure and function of bone are collectively determined by its extracellular matrix and intracellular contents. The bone matrix comprises both organic and inorganic components, with the organic fraction primarily consisting of type I collagen and the inorganic fraction predominantly composed of nano-hydroxyapatite (nHAp). Tropocollagen molecules initially assemble into collagen fibrils, which then aggregate to form collagen fibers [11]. Gaps between tropocollagen molecules contain charged amino acid residues that facilitate the nucleation and crystallization of amorphous calcium phosphate (ACP) at these sites [12, 13]. During crystallization, intrafibrillar gaps are initially filled with HAp to create plate-like structures aligned along the longitudinal axis, leading to intrafibrillar mineralization. Subsequently, further mineralization takes place on the surface of collagen fibrils to achieve extrafibrillar mineralization (Figure 2A) [14]. Both intrafibrillar and extrafibrillar mineralization are indispensable to the mechanical support of bone [15, 16].

Conversely, the cellular components of bone (osteoblasts, osteoclasts and osteocytes) are primarily involved in bone healing, which can be broadly divided into the inflammatory, bone-forming and remodeling phases (Figure 2B). Accumulated macrophages at the injury site modulate early anabolic activities to promote angiogenesis and osteogenesis during the inflammatory phase [17, 18]. In the subsequent bone-forming phase, mesenchymal stem cells (MSCs) are driven by the increased stiffness of the hematoma to commit to osteogenic lineage differentiation, which largely determines the eventual outcome of healing [19]. Recent studies have also highlighted that tissue stiffness and elasticity undergo continuous changes during remodeling [20, 21]. Overall, bone healing is governed by the evolving mechanical and biological microenvironments, underscoring the need for therapeutic strategies that integrate cellular regulation and microenvironmental modulation.

### Biomineralization

The principles of bone biomineralization are likewise highly complex, and many mechanisms remain to be fully elucidated. Various NCPs in the bone matrix play vital roles in collagen mineralization by both controlling crystallite morphology and stabilizing the amorphous phase [24, 25]. Nudelman *et al.* [26] also discovered that intrafibrillar mineralization occurred specifically within gaps of high positive net charge, revealing collagen’s active role in the process. Murshed [27] indicated that circulating phosphates (Pi) play a more prominent role than calcium ions (Ca^2+^) in the regulation of bone mineralization, and these bioactive Pi are present not only in the bone matrix but also within osteoblasts [28]. Therefore, bone mineralization is not merely a process of mineral deposition within the bone matrix; it also occurs intracellularly.

**Figure 3:**
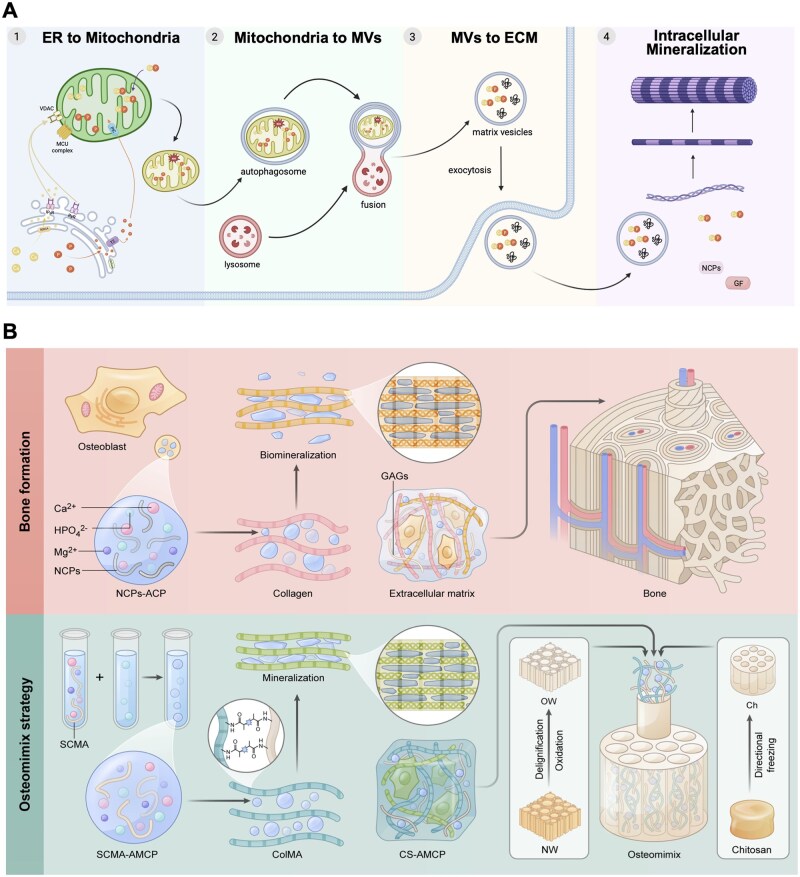
Overview of biomineralization and biomimetic mineralization. (**A**) Schematic illustration of bone biomineralization, which proceeds sequentially from intracellular mineralization to matrix mineralization. ER is for endoplasmic reticulum, and ECM is for extracellular matrix. Created with BioRender.com; reproduced with permission from Ref. [32], Copyright 2023, Elsevier; reproduced with permission from Ref. [46], Copyright 2025, American Chemical Society. (**B**) Inspirations from the multiscale hierarchical structure of native bone and the process of bone biomineralization provide design strategies of BMBs; reproduced with permission from Ref. [45], Copyright 2025, Wiley-VCH.

Mineralization initiates within the endoplasmic reticulum, where calcium and phosphate clusters form and eventually assemble into autolysosomes containing rich ACP precursors [29–31]. The vesicles, also known as matrix vesicles, are discharged into the bone matrix and subsequently participate in collagen mineralization (Figure 3A) [32, 33]. As a subtype of extracellular vesicles, matrix vesicles (MVs) are likewise affected by the mechanical properties of the bone matrix. Lenzini *et al.* [34] demonstrated that stress relaxation of the matrix relieves EVs from the escape cages formed by polymer networks, potentially accelerating the transport of mineralization precursors [35]. Traditionally, it has been assumed that the mineralization precursors reach their target sites via passive diffusion. Raguin *et al.* [36], however, reported that they can also be rapidly and precisely delivered through the interlinked nanochannels between collagen fibrils.

Once the mineralization precursors are delivered to the target sites, matrix mineralization occurs. Multiple hypotheses have been proposed to explain its underlying mechanism. Electrostatic attraction theory suggests that negatively charged NCPs facilitate the formation of negatively charged precursor–protein complexes, thereby enabling minerals to enter collagen via electrostatic attraction [37]. Electroneutrality/osmotic equilibrium balance theory further reveals that the osmotic pressure between the intra- and extrafibrillar spaces provides a long-range driving force for ACP to infiltrate collagen fibrils [38, 39]. Interfacial energy guided mineralization emphasizes that the confined gap geometry reduces the total energy barrier for HAp nucleation [40]. Other theories include size exclusion [41, 42], which emphasizes the role of NCPs smaller than 40 kDa, and collagen/apatite self-assembly [43], which highlights the predominant role of collagen in the process. Despite these efforts, none of these theories fully accounts for all phenomena observed in matrix mineralization [44].

The aforementioned studies have not only clarified many aspects of bone biology but also inspired further biomimetic design of BMBs. A recent study from our group has proposed a multidimensional biomimetic strategy to enhance bone regeneration by integrating a self-mineralized hydrogel with oriented bulk scaffolds. This strategy enabled the regulation of multiple signaling pathways, such as TGF-β, Wnt and cGMP–PKG and thereby achieved both bone regeneration and angiogenesis (Figure 3B) [45]. It seeks to leverage natural bone structure and biomineralization by employing BMBs to support the entire course of bone healing.

## Biomimetic mineralization strategies

To engineer BMBs that approximate the structure and function of native bone, multiple mineralization strategies have been explored. Among them, the wet chemical method remains one of the most established approaches. By blending or diffusion, the technique enables rapid and controllable HAp deposition [47, 48], although the heterogeneous nature of nucleation frequently leads to nonuniform mineral distribution. To address this limitation, Wu *et al.* [49] applied a ‘sequential mineralization’ protocol in which repeated cycles of the process improved homogeneity and performance of BMBs. Simulated body fluid (SBF) soaking represents a more biomimetic alternative, as it provides a physiologically ion-rich environment conducive to uniform mineral formation [50]. But the intrinsic kinetics are slow. Although concentrated SBF can accelerate mineralization, approximately 36 h are still required to form heterogeneous lamellar structures before supersaturation induces the formation of undesirable phases [50, 51].

Numerous novel biomimetic mineralization strategies have also been developed with the introduction of functional polymers. They broadly include the widely adopted PILP mineralization, enzyme-induced mineralization and the stabilizing effect of mineralization templates. These methods introduce specific molecular components, such as poly(acrylic acid) (PAA), the ALP enzyme, bovine serum albumin (BSA) and polyaspartic acid (pAsp). When polymers are linked to templates, the templates acquire bioactivity that enables them to regulate their own mineralization [52]. A significant advantage of this category lies in its ability to precisely modulate mineral nucleation and growth, potentially facilitating intrafibrillar mineralization [53]. Due to the diverse properties of introduced molecules, the experimental conditions can also become stringent. For instance, ALP-mediated mineralization is typically performed at a temperature of 38°C [54]. Conversely, methods collectively termed as spontaneous mineralization emphasize a ‘one-pot’ synthesis approach. This involves incorporating biomaterials or material precursors alongside mineralization agents during preparation [55]. While this significantly reduces the number of preparation steps, it imposes higher requirements on the composition of BMBs. Consequently, optimizing the operational conditions, required time and mineralization outcomes of existing biomimetic protocols remains a focus of ongoing research.

As understanding of biomineralization mechanisms has become more comprehensive and in-depth, researchers have, in recent years, proposed several novel biomimetic mineralization strategies with substantial potential for fabricating BMBs. Studies have shown that collagen possesses intrinsic piezoelectricity. It produces negative charges when compressed and positive charges when stretched, endowing bone with piezoelectric responsiveness. A growing body of research has investigated the application of piezoelectricity in bone regeneration. This interest stems from its capacity to convert mechanical stimuli—originating from daily activities or noninvasive inputs—into endogenous electrical signals. Such electromechanical coupling directly encourages intracellular mineralization, a part of cell-dependent mineralization and supports the development of a pro-osteogenic bioelectrical microenvironment [56]. Subsequently, Kwon and Cho [57] proposed that piezoelectricity can guide the mineralization of collagen. This concept motivated the use of ultrasound to induce rapid mineral deposition on piezoelectric-responsive poly(L-lactic acid) (PLLA) modified with polydopamine (PDA). However, detailed operational parameters and underlying mechanisms were not clarified [58]. In contrast, Orrego *et al.* [59] achieved piezoelectricity-induced mineralization of poly(vinylidene fluoride) (PVDF) during SBF soaking, thereby establishing a direct link between piezoelectricity and mineralization. Under cyclic compressive loading of 5 N, the material generated an electrical charge of up to ∼2.5 pC. This stimulation redistributed electric dipoles within the matrix and increased the surface negative charge density. As a result, the continuous attraction of metal ions from the surrounding environment facilitated a ‘self-mineralization’ process, accompanied by an approximately 180% increase in Young’s modulus. Nonetheless, experimental approaches that directly utilize piezoelectricity for biomimetic mineralization have yet to be systematically investigated.

In bone, mineralized collagen fibers serve as the fundamental structural units and directly support its mechanical resilience. Recently, our group highlighted the pivotal role of mechanical force in collagen formation, assembly and mineralization, which directly links mechanical stimulation with mineralization [35]. Mechanistically, cyclic loading and similar stimuli regulate both the production and cargo-loading of MVs, thereby regulating the availability of minerals. At the same time, mechanical cues help overcome the confinement of the extracellular matrix (ECM) on ACP diffusion. This facilitates the smooth transport of nucleation precursors into the gap zones within collagen fibrils and optimizes mineral delivery. Du *et al.* [60] applied fluid shear stress of 1.0 Pa with alternating 1-h stimulation and 1-h rest to mimic physiological loading patterns of collagen. In a subsequent study, the same group developed an *in vitro* model of cyclic compressive stress with a strain threshold of 1.5% at 0.5 Hz to approximate the frequency of normal human walking. The resulting mineralized collagen exhibited an enhanced compressive modulus of approximately 2.5 MPa [61]. These strategies resulted in smooth, highly ordered and well-aligned intrafibrillarly mineralized collagen, with promising potential as BMBs. Taken together, ongoing advances in biomineralization mechanisms are driving the refinement of mineralization strategies, thereby enabling the development of BMBs with simplified fabrication, improved mechanical compatibility, enhanced bioactivity and superior healing outcomes (Table 1). Nevertheless, many emerging approaches still lack clearly defined mechanisms, and experimental efforts often focus on material fabrication rather than functional evaluation in bone regenerative contexts.

**Table 1:** Comparison of biomimetic mineralization strategies.

Strategy	Advantages	Disadvantages	Reference(s)
Wet chemical method	Well established; Certain controllability through experimental conditions	Nonuniform mineral distribution	[47–49]
SBF soaking	Improved mineral distribution; Physiological similarity of mineral resources	Intrinsically slow kinetics	[50, 51]
Polymer-induced			
PILP	Molecular precision; Optimized mineral distribution; Success of intrafibrillar mineralization	Limited stability	[62–64]
Enzyme-induced	Faithful mimicry of biomineralization	Stringent conditions	[54, 65]
Functional template	Oriented mineral alignment; Highly designable	Lack of standardized protocols	[66–68]
Spontaneous mineralization	Simple operation	Restricted applicability	[55, 69, 70]
External stimulation			
Photo-induced	Uniform mineral distribution; Precise control over mineralization	High production cost; Limited yield	[69, 71, 72]
Piezoelectrically induced	Retaining piezoelectric properties	Potential cytotoxicity of introduced piezoelectric particles	[57, 58, 73, 74]
Mechanically induced	Influence on both scaffold matrix and mineralization precursors	Complex equipment	[35, 60, 61]

## Classification of BMBs

### Biopolymers

Biopolymers are long-chain macromolecules composed of repeating monomeric units. They can be either naturally derived (e.g. collagen, gelatin, silk fibroin, cellulose, chitin, chitosan) or synthetically produced [e.g. polycaprolactone, polylactic acid, poly(lactic-co-glycolic acid)]. Owing to their biocompatibility, excellent biodegradability and nontoxicity, they can be used to construct scaffolds that support bone regeneration.

#### Collagen-based materials

**Figure 4:**
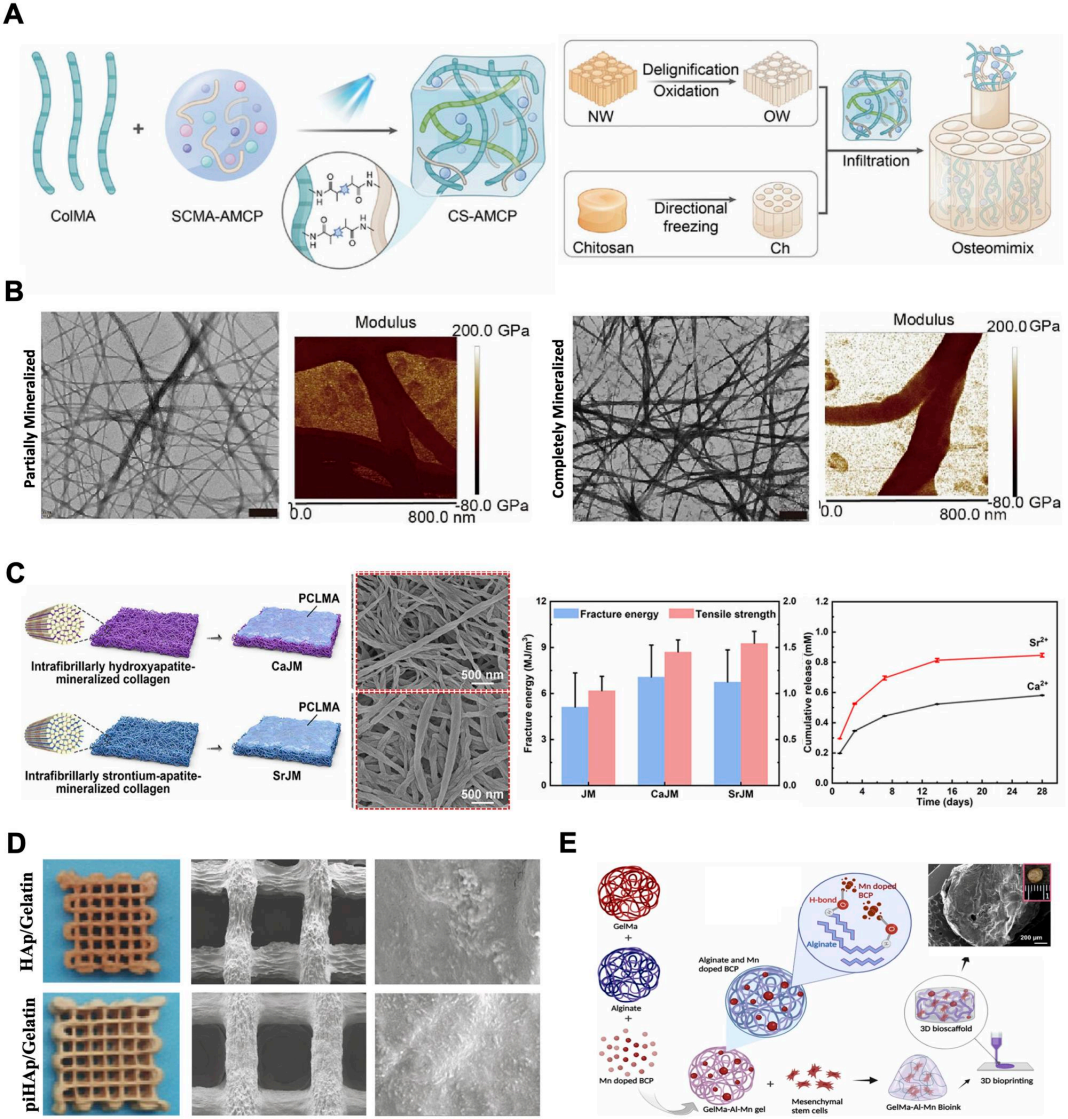
Design strategies and characterization of collagen- and gelatin-based BMBs. (**A**) Fabrication of a multidimensional BMB, osteomimix, in which collagen plays a pivotal role; reproduced with permission from Ref. [45], Copyright 2025, Wiley-VCH. (**B**) Surface morphology of intrafibrillarly mineralized collagen fibrils and their mechanical strength; reproduced with permission from Ref. [96], Copyright 2023, Elsevier. (**C**) Janus GBR membranes fabricated from collagen fibrils mineralized with distinct components, along with their surface morphology, mechanical properties and ion release characteristics; reproduced with permission from Ref. [97], Copyright 2024, American Chemical Society. (**D**) Morphology of a 3D-printed *in situ*–mineralized gelatin scaffold; reproduced with permission from Ref. [98], Copyright 2022, Elsevier. (**E**) Formation and morphology of a mineralized GelMA scaffold; reproduced with permission from Ref. [99], Copyright 2023, American Chemical Society.

Mineralized collagen fibrils are the fundamental building blocks of natural bone. Due to their structural and compositional similarity to native bone, collagen-based materials have naturally emerged as a popular choice in bone regeneration (Figure 4A).

In natural bone, collagen is composed of a fundamental repeating Gly–X–Y tripeptide unit, where glycine invariably occupies the first position, proline is frequently found at the X position, and hydroxyproline commonly occupies the Y position [75]. These repeating units assemble into three polypeptide chains that intertwine to form a triple helix [76]. Subsequently, multiple tropocollagen molecules align in a parallel, highly ordered fashion, producing the characteristic D-band with an approximate length of 67 nm [77]. With excellent biocompatibility, strong cell adhesion and reliable osteoconductivity, collagen and its engineered forms have emerged as key materials in BTE [78]. Nevertheless, native collagen exhibits inherently limited mechanical strength and undergoes rapid biodegradation, so it is frequently combined with inorganic constituents to attain the desired performance.

In general, collagen-based materials can be fabricated using a variety of techniques, such as electrospinning [79], layer-by-layer assembly [80], freeze-drying [81] and 3D printing [82]. In contrast to pure collagen and extrafibrillarly mineralized collagen scaffolds, intrafibrillarly mineralized collagen-based scaffolds generate megapascal contractile stresses [83] and more effectively promote both *in vitro* osteogenic differentiation and *in vivo* bone healing [84]. These superior mechanical and biological performances have made intrafibrillar mineralization a preferred strategy in recent BMB designs (Figure 4B). Wu *et al.* [85] broadly categorized the substances used to regulate intrafibrillar mineralization into three groups: (i) NCPs, such as osteopontin (OPN) and osteocalcin (OCN); (ii) NCP analogs, including pAsp, PAA and polyampholyte carboxymethyl chitosan (CMC); and (iii) bioactive small molecules, such as amino acids, citrate and fluoride.

Collagen-based BMBs can be fabricated in various forms, each with distinct advantages. For example, collagen microspheres feature controllable particle sizes, making them potential carriers for drugs or growth factors. In contrast, absorbable collagen membranes, typically derived from bovine tendons or porcine peritoneum, function as soft tissue barriers in guided bone regeneration (GBR) (Figure 4C). Collagen hydrogels are more adaptable to the shape of defects. Their mechanical strength and degradation rate can be tuned by adjusting the degree of crosslinking. Additionally, collagen sponges, with their porous structure, provide both hemostatic function and support for angiogenesis [78, 86]. Taken together, these distinct structural variations enable collagen-based BMBs to be tailored to different clinical scenarios of bone defects.

#### Gelatin-based materials

Gelatin is a natural polymer derived from the hydrolysis of collagen. They share similar extraction pathways, structural features and biological functions. As an ECM analog, gelatin possesses a characteristic arginine–glycine–aspartic acid (RGD) sequence that facilitates cell adhesion, proliferation and differentiation, which makes it a promising candidate [87]. In addition, it can be fabricated into various forms—such as scaffolds, hydrogels and nanoparticles—via techniques including 3D printing, electrospinning and freeze-drying (Figure 4D). This allows gelatin to adapt to bone defects with diverse geometries. Gelatin also has inherent limitations as a repair material, such as low mechanical strength and high degradability, which restrict its independent use as a scaffold [88]. However, gelatin methacrylate (GelMA) has gained considerable attention for its favorable photocrosslinking properties, positioning it as a promising platform for BMBs (Figure 4E) [89].

Beyond conventional inorganic ceramics, a range of emerging materials has been integrated into gelatin-based systems, among which nanoclay has attracted notable interest. Nanoclay contains multiple elements that are beneficial for mineralization, such as sodium, aluminum, zinc and magnesium [90]. Posada-Lotero *et al.* [91] incorporated montmorillonite (MMT) nanoclay into HAp/gelatin composites to effectively enhance the overall mechanical strength and toughness. Lukin *et al.* [92] also incorporated a saponite-derived nanoclay into the gelatin network. They found that the nanoclay not only enhanced the mechanical properties of materials but also exhibited mineralization-inducing and osteogenesis-promoting capabilities. However, recent studies have highlighted potential challenges that nanoclay may trigger stronger cytotoxic responses and exhibit weaker mineralization compared with nHAp [93, 94]. In addition, phenolic compounds represent another important direction for modification, as they can confer antibacterial properties to materials while enhancing osteoblast activity. For instance, Hobbi *et al.* [95] modified gelatin-based scaffolds with phloridzin—a polyphenolic compound extracted from apple flesh—to induce mineralization, and the resulting material demonstrated excellent bone regeneration.

#### Silk fibroin-based materials

**Figure 5:**
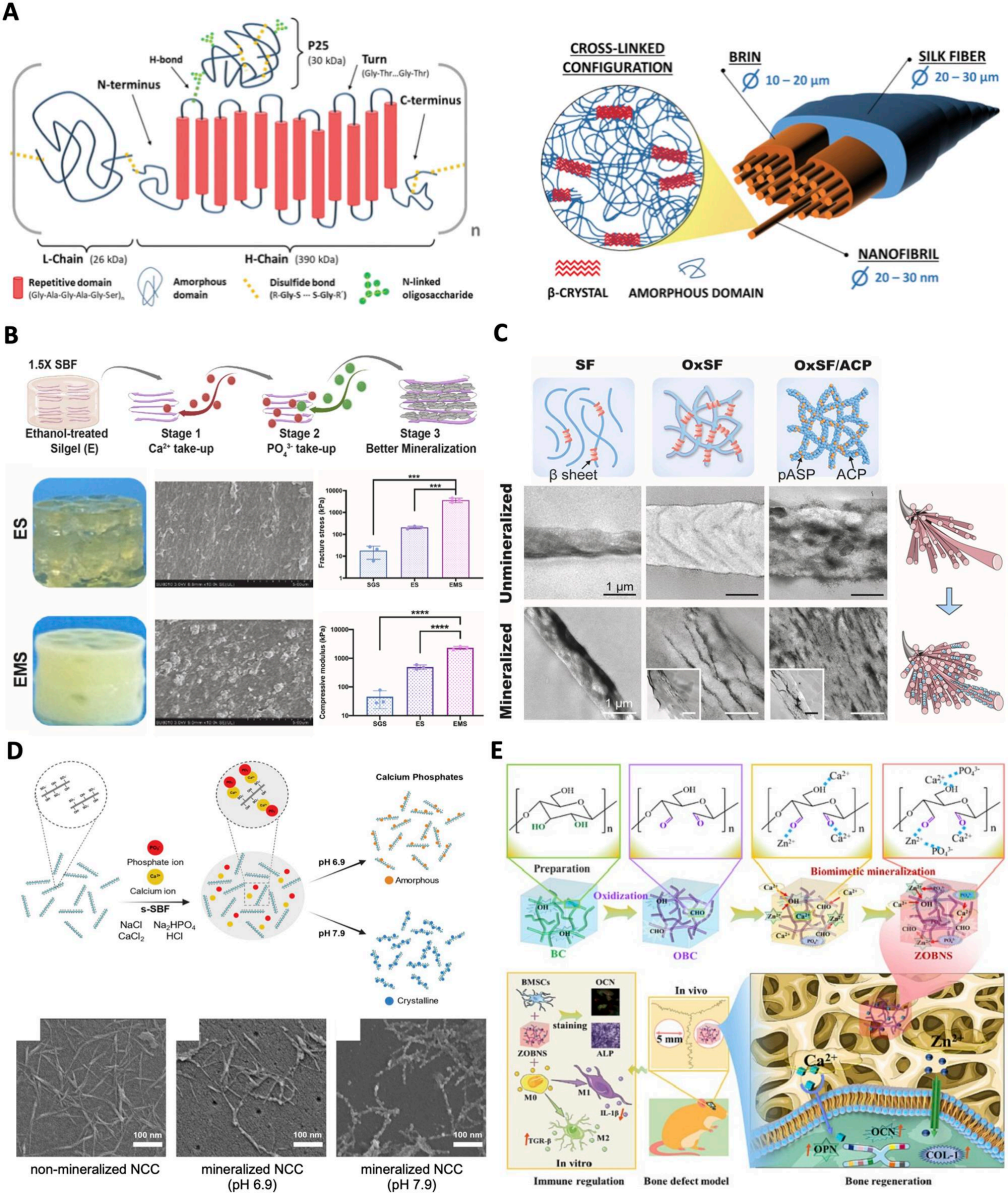
Design and characterization of silk fibroin- and cellulose-based BMBs. (**A**) Schematic illustration of the folding of major silk fibroin proteins and their aggregation into compact structures; reproduced with permission from Ref. [100], Copyright 2022, Wiley-VCH. (**B**) Schematic illustration of the mineralization of methacrylated silk fibroin, along with its morphology and mechanical properties; reproduced with permission from Ref. [118], Copyright 2024, Elsevier. (**C**) Morphology of a mineralized silk fibroin–based composite hydrogel; reproduced with permission from Ref. [108], Copyright 2024, Elsevier. (**D**) Mineralization and morphology of NCCs; reproduced with permission from Ref. [119], Copyright 2022, Elsevier. (**E**) Fabrication of a calcium (Ca^2+^)/zinc (Zn^2+^)-mineralized cellulose-based scaffold; reproduced with permission from Ref. [117], Copyright 2024, Elsevier.

Silk fibroin (SF) is a fibrous protein primarily produced by silkworms and spiders. While sharing certain homologies with collagen, SF adopts a highly ordered β-sheet structure rather than a triple helix [100]. This unique structure endows SF with superior mechanical strength and excellent biocompatibility. Moreover, non-mulberry SF contains RGD sequences that mediate cell adhesion [101], further underscoring its potential as a promising material for BTE (Figure 5A). Some studies suggest that low-molecular-weight degradation products of SF induce mild inflammatory responses. Still, given that other research disputes this [102, 103], such inflammation may not indicate poor biocompatibility [104].

SF can be processed into hydrogels, sponges, fibers, microspheres and other biomaterial forms with tunable properties. Although the intrinsic β-sheet structure can partially improve the mechanical performance, incorporating inorganic particles or polymers is still a more popular strategy (Figure 5B) [105]. For example, Kundu *et al.* [106] prepared mineralized SF hydrogels by inducing HAp deposition with SBF treatment, thus achieving strong mineralization and osteogenic potential. Similarly, introducing pAsp into SF scaffolds controls calcium phosphate deposition and results in mineralized scaffolds with enhanced *in vitro* osteogenesis [107]. Zhu *et al.* [108] further fabricated a composite SF-based hydrogel in which oxidized silk fibroin (OxSF) served dually as a mineralization template and an ACP stabilizer. This innovative design promoted biomineralization and achieved exceptional osteogenic performance (Figure 5C).

#### Cellulose-based materials

As the most abundant natural polymer in the world, cellulose is widely found in plants and offers advantages such as easy availability, low cost, good biocompatibility and high tunability. Its β-1,4-linked glucose residues assemble into extended chains that resemble the fibrous architecture of collagen in bone, contributing to mechanical compatibility within the bone microenvironment [109, 110]. Collectively, these features enable cellulose and its derivatives to become promising candidates for BTE. However, its poor biodegradability limits the biomedical application. Thus, *in vivo* degradation of cellulose often requires optimization through hydrolysis, enzymatic degradation, chemical oxidation (with oxidizing agents) and physical degradation (mechanical loading and wear) [111].

Based on its morphology, nanocellulose can be classified into three main types: nanofibrillated celluloses (NFCs) with good flexibility, nanocrystalline celluloses (NCCs) with high crystallinity and bacterial celluloses (BCs) synthesized by bacteria [112]. NFC exhibits good thermal stability, but its mechanical properties are relatively poor [113]. NCC, solely consisting of crystalline regions, possesses high mechanical strength to reinforce BTE scaffolds (Figure 5D) [114]. Meanwhile, BC possesses remarkable biocompatibility and is used in diverse biomedical applications [115]. Cellulose and its derivatives can also serve as organic templates for biomimetic mineralization. For example, Wu *et al.* [116] used carboxymethyl NFCs as both mineralization template and an amorphous calcium carbonate stabilizer to obtain high-strength and high-toughness materials with osteogenesis potential. Similarly, Luo *et al.* [117] successfully induced deposition of calcium/zinc-hydroxyapatite (HAp) on BC-based scaffolds with significant osteogenic capability under simulated physiological conditions (Figure 5E).

#### Chitin-based materials

**Figure 6:**
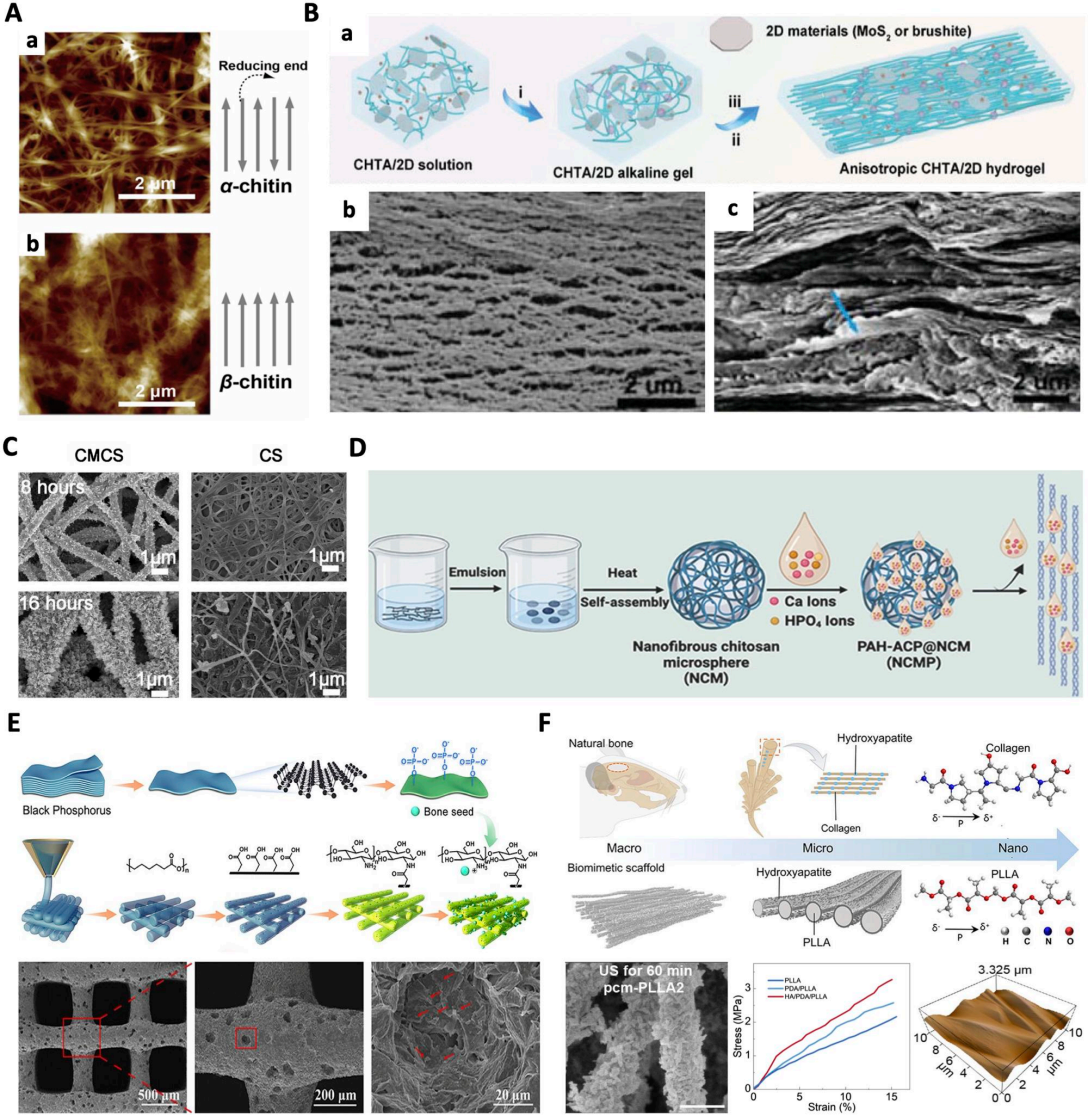
Preparation and performance of chitin-, chitosan- and synthetic polymer-based BMBs. (**A**) The microstructure of α-chitin and β-chitin; reproduced with permission from Ref. [123], Copyright 2022, American Chemical Society. (**B**) Design strategy and morphology of an anisotropic biomineralized chitin-based material; reproduced with permission from Ref. [127], Copyright 2022, Wiley-VCH. (**C**) SEM images of carboxymethyl chitosan and chitosan nanofibers immersed in 5xSBF for mineralization; reproduced with permission from Ref. [50], Copyright 2018, Elsevier. (**D**) Illustration of a chitosan-based microsphere used in bioinspired mineralization; reproduced with permission from Ref. [135], Copyright 2025, Elsevier. (**E**) Fabrication and morphology of a 3D-printed black phosphorus/PCL scaffold; reproduced with permission from Ref. [145], Copyright 2024, Wiley-VCH. (**F**) Design and characterization of a mineralized PLLA scaffold; reproduced with permission from Ref. [58], Copyright 2024, Elsevier.

Chitin is the second most abundant polymer present in nature. Owing to bioactivity, biocompatibility, biodegradability and nontoxicity, it has attracted considerable attention in BMBs [120]. Based on its microstructure, the chitins commonly used in biomedical applications are α-chitin and β-chitin. α-Chitin, which is more abundant in nature, is mainly derived from fungal cell walls and exoskeletons of arthropods, whereas β-chitin is less common and can be found in squid pens and cuttlebones [121]. The molecular chains of β-chitin are arranged in parallel, which weakens hydrogen bonding between intermolecular sheets and consequently produces a looser structure (Figure 6A) [122]. Recently, Bai *et al.* [123] proposed nanochitin, the fundamental structural unit of chitin, as an alternative material with similar reactivity and a greener, more sustainable preparation, highlighting a novel direction for chitin-based BMBs.

Based on these favorable properties, Kawata *et al.* [124] employed β-chitin nanofibers as a template to fabricate mineralized chitin hydrogels with enhanced mechanical performance. Arun Kumar *et al.* [125] used nHAp to biomimetically mineralize α-chitin-based composites, which not only increased the elastic modulus of the material but also improved its ability to promote the osteogenic differentiation of MSCs. Furthermore, Xing *et al.* [126] incorporated HAp/chitin into collagen scaffolds to enhance their compressive strength and further verified their *in vivo* osteogenic potential. Our group has also developed chitin–tannic acid/brushite hydrogels. In this design, the high stiffness of chitin nanofibers was retained while the elastic modulus and osteogenic differentiation ability were improved through brushite-based biomimetic mineralization. These features collectively endow the material with promising potential for BTE (Figure 6B) [127].

#### Chitosan-based materials

Due to strong intramolecular hydrogen bonding, chitin, particularly the more abundant α-chitin, possesses a dense and stable structure that limits its processability in biomaterial fabrication [128]. In contrast, its deacetylated derivative, chitosan, not only shares similar biological properties but also exhibits hydrophilicity and polycationic properties [129]. More importantly, the structure of chitosan closely resembles that of glycosaminoglycans (GAGs), which facilitate cell adhesion and protein binding [130]. Once the organic templates are prepared, biomimetic mineralization techniques are typically applied to enhance the inherently weak mechanical properties of natural chitosan [131].

Numerous studies have employed chitosan-based scaffolds to construct BMBs with excellent *in vitro* and *in vivo* osteogenic performance (Figure 6C). Zhang *et al.* [132] developed mineralized scaffolds of chitosan–gelatin/HAp, which significantly enhanced MSC differentiation. Similarly, Zou *et al.* [133] fabricated nHAp/alginate/chitosan scaffolds that not only promoted BMSC osteogenic differentiation but also exhibited potential for drug delivery. Olza *et al.* [134] further reinforced chitosan scaffolds with biomimetically mineralized chitin nanocrystals to improve osteoinductivity. Beyond this, our group has also explored the use of chitosan microspheres to mimic matrix vesicle-mediated ACP delivery and eventually achieved intrafibrillar mineralization of collagen (Figure 6D) [135]. Overall, these studies highlight both the structural versatility and functional tunability of chitosan-based BMBs.

#### Synthetic polymer-based materials

Synthetic polymers, produced through chemical or biosynthetic routes, are extensively employed in BTE owing to their biocompatibility, biodegradability and structural tunability with functional group modification. Their versatility allows them to mimic the organic phase of bone and to be tailored to specific requirements. Nevertheless, synthetic polymers typically suffer from limited bioactivity, absence of cell-recognition sites and poor osteoconductivity, thus requiring the integration of bioactive components [136–138]. Among various synthetic polymers, aliphatic polyesters are most commonly studied, including polycaprolactone (PCL), polylactic acid (PLA), poly(lactic-co-glycolic acid) (PLGA) and polyglycolic acid (PGA) (Figure 6E) [139]. For instance, PLA/HAp composite scaffolds not only exhibit favorable mechanical properties but also promote bone regeneration [140]. PLLA scaffolds can be further modified with bioactive glass to enhance the mechanical strength and facilitate HAp deposition (Figure 6F) [141]. However, recent studies suggest that the degradation of specific synthetic polymers can produce acidic or poorly crystalline by-products that provoke inflammation [142, 143]. In contrast, the slow or incomplete resorption of others may lead to chronic inflammatory responses [144]. These findings underscore the need for more rigorous evaluation of their long-term biosafety. Given the wide range of polymers introduced as materials for BMBs, representative examples are summarized along with their fabrication techniques and outstanding properties in Supplementary Table S1.

### Bioactive ceramics

**Figure 7:**
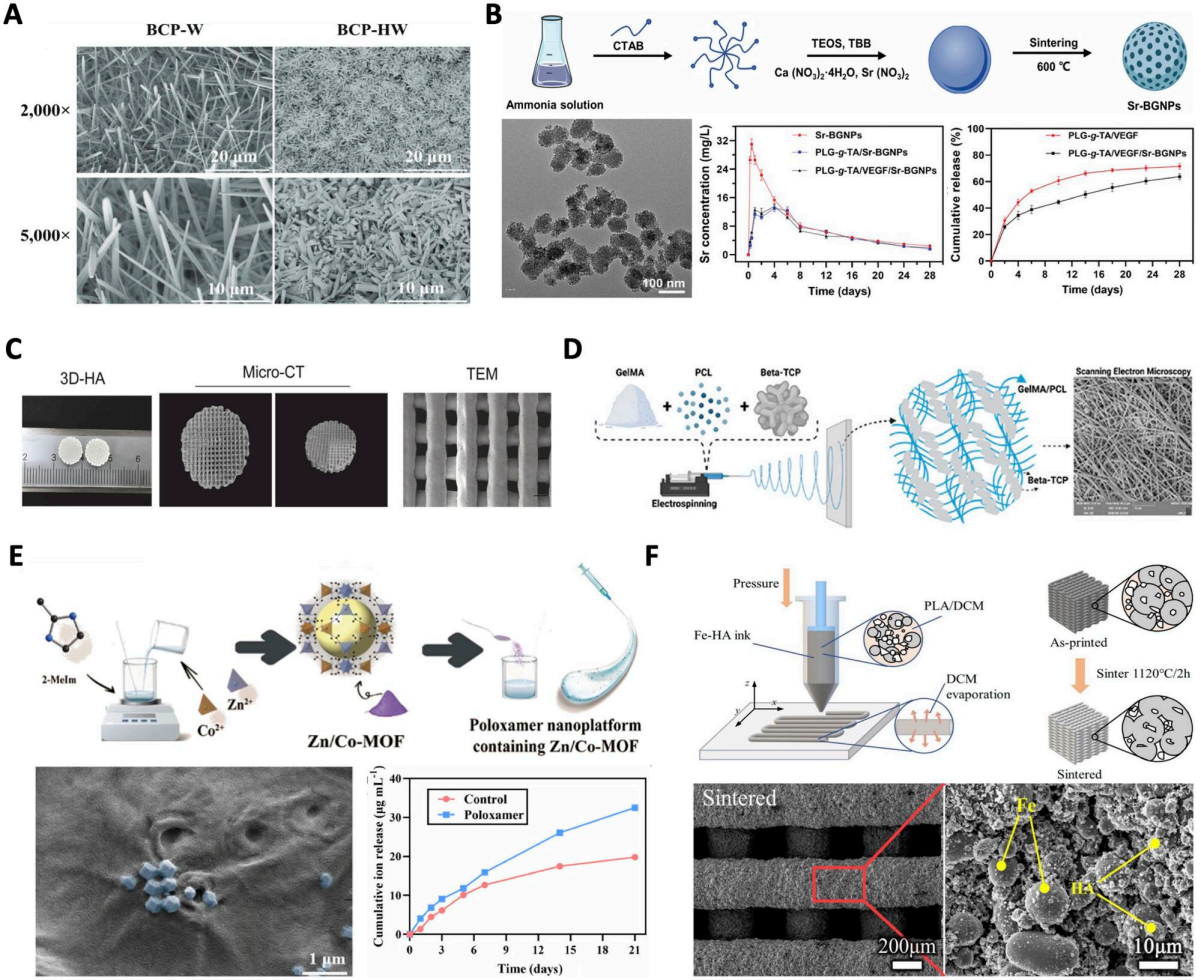
Construction and characterization of bioactive ceramics and metals in BMBs. (**A**) Morphology of two distinct BCP ceramics; reproduced with permission from Ref. [173], Copyright 2022, Springer Nature. (**B**) Fabrication, morphology and ion release behavior of bioactive glass particles; reproduced with permission from Ref. [174], Copyright 2024, Wiley-VCH. (**C**) Morphology of a 3D-printed HAp scaffold; reproduced with permission from Ref. [158], Copyright 2022, Elsevier. (**D**) Illustration of a GelMA/PCL composite membrane reinforced with β-TCP; reproduced with permission from Ref. [175], Copyright 2023, American Chemical Society. (**E**) Fabrication, morphology and release behavior of zinc/cobalt bimetallic metal–organic frameworks (Zn/Co-MOFs); reproduced with permission from Ref. [176], Copyright 2025, Wiley-VCH. (**F**) Fabrication and morphology of a 3D-printed Fe–HAp scaffold; reproduced with permission from Ref. [177], Copyright 2022, Elsevier.

Bioactive ceramics represent a significant class of ceramic materials for bone replacement and regeneration and can be broadly categorized into calcium phosphate ceramics and bioactive glasses [146]. Notably, calcium phosphate ceramics encompass HAp and its related phases. As the principal inorganic constituent of bone and a standard product of biomimetic mineralization, HAp has been extensively incorporated into polymer-based composites to enhance bioactivity [147]. Other calcium phosphates, such as tricalcium phosphate (TCP), ACP and biphasic calcium phosphate (BCP), have also attracted significant attention (Figure 7A). TCP exhibits excellent biocompatibility but undergoes rapid *in vivo* resorption, which may compromise sustained osteoconductivity [148, 149]. ACP, which is regarded as a transient precursor during bone mineralization, readily transforms into HAp under physiological conditions [150]. In comparison, BCP, composed of both HAp and β-TCP, integrates the chemical stability of HAp with the degradability of β-TCP, thereby enabling precise modulation of dissolution and stability [151].

Bioactive glasses, conversely, are glass-based materials that exhibit osteogenic, angiogenic and antibacterial properties *in vivo* [152]. They can be broadly classified into silicate, borate and borosilicate glasses [153, 154]. One of the defining features of bioactive glasses is their controlled dissolution behavior, which releases bioactive ions while providing mechanical support. This dual function not only promotes bone formation but also facilitates the integration of soft and hard tissues [155]. For example, Li *et al.* [156] demonstrated that the release of calcium, silicon and phosphates from bioactive glass could upregulate osteogenic markers such as Runx2 and Col-I (Figure 7B).

In bone regeneration, bioactive ceramics can be directly fabricated into scaffolds for bone defect repair (Figure 7C), where calcium phosphate ceramics have particularly demonstrated the superior osteoinductive and angiogenic potential [157]. Gao *et al.* [158] used HAp scaffolds loaded with BMSC-derived exosomes for bone defect healing, while Zhao *et al.* [159] enhanced HAp scaffolds with polyphenols to further promote new bone formation. But more commonly, bioactive ceramics serve as reinforcing phases to yield composites with improved mechanical properties and bioactivity (Figure 7D). Recently, whitlockite [Ca_18_Mg_2_(HPO_4_)_2_(PO_4_)_12_] has gained attention as a promising bioactive ceramic. When combined with HAp, it can alleviate the inhibitory effect of long-lasting implants by improving degradability [160, 161].

### Metals

Due to their superior mechanical properties, metallic materials are widely used in clinical practice, particularly for large-scale bone defects and severe skeletal injuries [162]. Common metals for bone regeneration include stainless steel and titanium, with porous titanium showing notable advantages for cell adhesion [163]. While metals provide unparalleled structural support compared with other single-material systems, their lack of *in vivo* degradability often leads to complications such as secondary surgery and infection. Incorporating bioactive metal ions into polymer-based scaffolds, therefore, offers a degradable alternative, as these ions bond with organic scaffolds to drive biomineralization and promote regeneration. The released metal ions can also exert antibacterial and anti-infective effects [164].

Metal-organic frameworks (MOFs), a class of organic–inorganic hybrid materials, have emerged as promising scaffold components in BTE due to their high specific surface area, customizable porosity and tunable frameworks, which collectively facilitate bone healing (Figure 7E) [165, 166]. Beyond scaffold applications, MOFs also show antibacterial properties and therapeutic potential for bone-related diseases [167, 168]. In parallel, the development of biodegradable metals [169] and their composites, particularly biodegradable metal matrix composites [170], further expands the scope of metals for bone regeneration (Figure 7F). For example, Zhao *et al.* [171] fabricated GelMA–bisphosphonate hydrogels to capture magnesium ions (Mg^2+^), thus forming a microsphere that exhibited significant osteogenic potential. Wang *et al.* [172] reported that titanium dioxide coated with Zn^2+^ can faithfully promote new bone ingrowth. Taken together, these studies highlight the importance of metal ions in enhancing the bioactivity, osteogenic potential and overall performance of BTE materials.

## Applications of BMBs in BTE

### Osteogenesis

Among all the functions of BMBs, the ability to promote osteogenesis is the most fundamental and critical. Such materials should not only mimic the composition and mechanical strength of natural bone but also possess bioactivity that stimulates endogenous osteogenic factors and pathways to achieve osteoconductivity, osteoinductivity and osteogenesis. Their chemical composition strongly influences the osteogenic potential of BMBs. Experiments have shown that HAp in materials can effectively activate the PI3K–Akt signaling pathway, thereby enhancing the osteogenic differentiation of BMSCs [178]. Similarly, Mg^2+^ also plays a pivotal role. Mg^2+^ deficiency inhibits the expression of osteogenic genes, whereas an increasing Mg^2+^ level promotes BMSC osteogenic differentiation via the Notch1 signaling pathway [179, 180]. Zhao *et al.* [171] developed GelMA-BP-Mg injectable hydrogel microspheres capable of releasing Mg^2+^ for up to 18 days, thus providing prolonged osteogenic promotion. Other metal ions such as zinc, cobalt and strontium can also be incorporated into BMBs as functional components to enhance osteogenesis [181–183].

**Figure 8:**
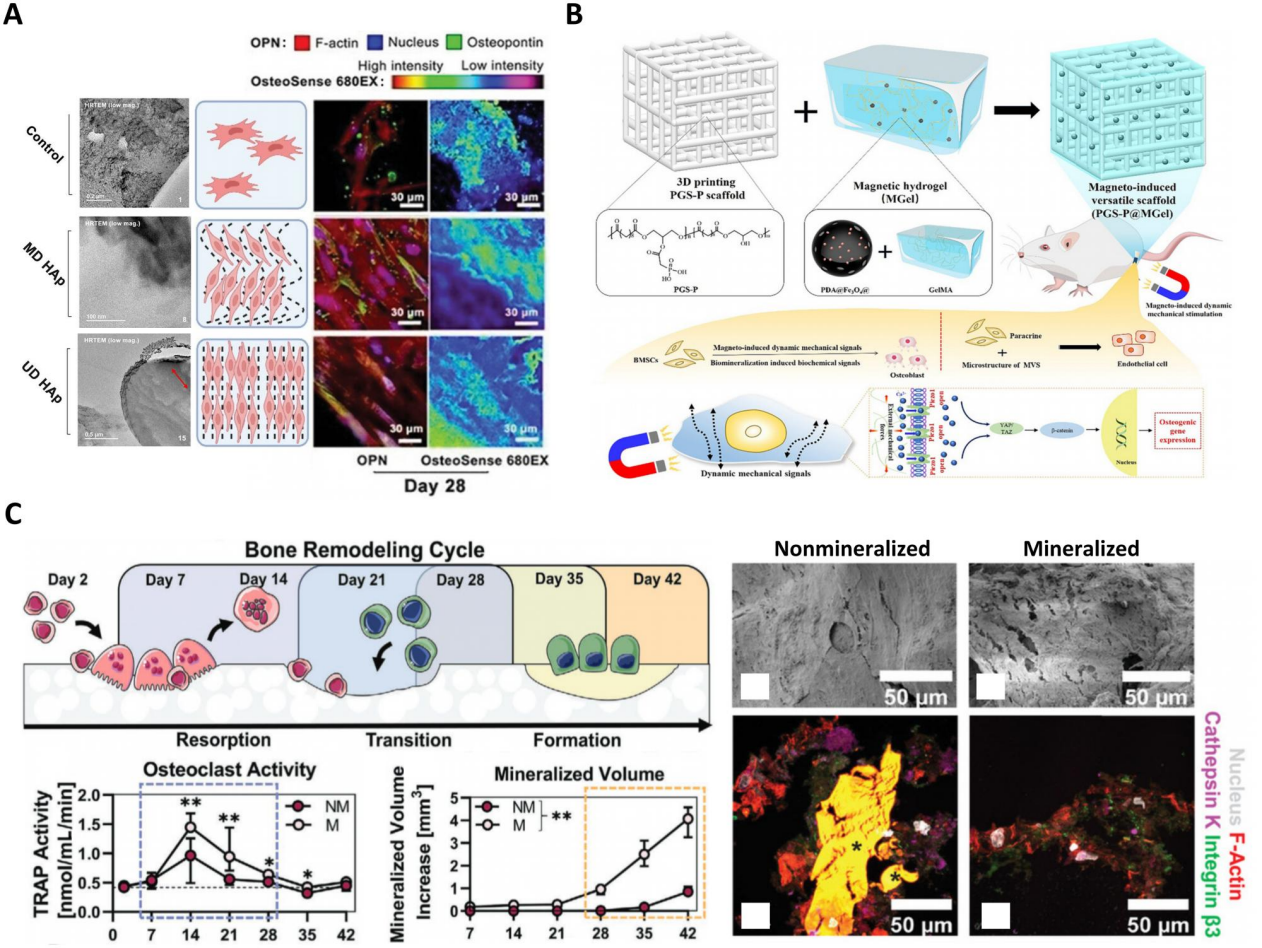
Applications and mechanisms of BMBs in osteogenesis. (**A**) Unidirectional (UD) HAp nanorods induce ordered collagen mineralization and enhance osteogenic potential compared with multidirectional (MD) HAp; reproduced with permission from Ref. [185], Copyright 2024, Wiley-VCH. (**B**) Schematic illustration of the preparation and application of a self-mineralized scaffold which can be mechanically stimulated under an external magnetic field; reproduced with permission from Ref. [70], Copyright 2025, Wiley‐VCH. (**C**) Mineralized silk fibroin scaffolds recapitulate the physiological bone remodeling process, from osteoclastic resorption to osteoblastic formation; reproduced with permission from Ref. [205], Copyright 2022, Wiley-VCH.

Factors influencing the osteogenic performance of BMBs extend beyond chemical composition to include structural properties [184]. Among these, anisotropic structure is widely recognized to promote bone formation. Our group has fabricated anisotropic biomimetic mineralized chitin–tannic acid/brushite hydrogels to mimic natural bone and found that such a structure increases new bone density by nearly 2.5-fold and bone volume fraction by almost 1-fold [127]. Similarly, Chen *et al.* [185] engineered unidirectionally aligned HAp nanorods that directed ordered collagen mineralization, enhanced cytoskeletal tension and activated the PI3K–Akt pathway (Figure 8A). Meanwhile, pore size also affects osteogenesis. Lv *et al.* [186] and Jiang *et al.* [184] both highlighted a pore size of 100–250 µm in chitin/HAp and chitosan/HAp scaffolds as favorable for osteogenesis. Studies on bioactive ceramics reported a broader effective range (100–900 µm) [187, 188], while work on metal alloys suggested an optimal window of 400–600 µm [189–191]. Although no consensus has been reached, the underlying mechanisms are well understood. Tiny pores restrict nutrient and oxygen diffusion, which promotes cell viability, whereas excessively large pores allow fibroblast infiltration that weakens scaffold integrity. Zhang *et al.* [192] also constructed polyhedral scaffolds that recapitulated the multi-porous architecture of trabecular bone, which more effectively activated PI3K–Akt signaling and promoted osteogenesis than conventional cross-over structures. These results collectively highlight the critical role of duplicating native bone architecture in achieving favorable osteogenic outcomes.

The mechanical microenvironment established by BMBs also contributes to osteogenesis. Wang *et al.* [193] identified Piezo1 as a mechanosensitive protein that directly senses loading to regulate bone remodeling and osteogenesis through YAP-mediated activation of the Wnt/β-catenin pathway [194]. The finding indicates that the mechanical properties of BMBs exert a decisive influence on osteogenesis outcomes. Other studies have shown that stem cells preferentially differentiate when cultured on substrates whose elasticity matches that of the target tissue [195], suggesting that BMBs should approximate the elastic modulus of bone. Considering that single materials often cannot meet such mechanical requirements, incorporating multiple inorganic components with biomimetic mineralization can effectively optimize the overall stiffness [196]. For example, Li *et al.* [197] achieved biomimetic mineralization of a collagen membrane using high-molecular-weight poly(acrylic acid) (HPAA). The resulting stiffer membranes significantly accelerated *in situ* bone regeneration by suppressing Hippo–YAP/TAZ signaling. It was further reported that materials with initially high stiffness that gradually soften promoted osteogenic differentiation of BMSCs, whereas porous low-stiffness materials favored chondrogenic differentiation [198]. This result underscores the spatiotemporal dynamics of mechanical cues in directing the fate of bone cells. These findings motivated Guo *et al.* [70] to develop a bone regeneration scaffold termed PGS-P@MGel, which combines self-mineralization capability and mechanical responsiveness. Its design centers on phosphorylated poly(glycerol sebacate), which continuously binds free Ca^2+^ to drive self-mineralization and on magnetically responsive PDA@Fe_3_O_4_ nanoparticles that deliver mechanical stimulation under an external magnetic field, ultimately enhancing vascularized bone regeneration (Figure 8B).

*In vivo* bone healing is a dynamic process that involves both osteoclastic and osteoblastic activities. While studies often regarded inhibition of osteoclast function as beneficial, accumulating evidence indicates that moderate osteoclastic activity can actually support osteoblastic function [199]. Preliminary observations by Robin *et al.* [9] suggested that such enhancement may arise from elevated bone remodeling activity mediated by osteoclasts. Indeed, active osteoclasts can secrete cytokines, such as TGF-β, to enhance osteoblast activity and outcomes [200]. Their small extracellular vesicles (SEVs) also contain RANK, which initiates osteoblast differentiation at early stages [201]. Given these clues, some researchers propose that if an implant undergoes initial osteoclastic resorption followed by osteoblast-mediated bone formation, the resulting bone will exhibit superior performance [202]. Consistently, Zhang *et al.* [203] confirmed that osteoclasts can effectively enhance ectopic bone formation. Some material studies further illustrate this dual role. Osteoclast behavior on intrafibrillarly mineralized collagen scaffolds was impaired due to their porous architecture and lack of RGD motifs [204]. In contrast, mineralized silk fibroin scaffolds allowed osteoclast adhesion and bone resorption, reproducing the entire bone remodeling process (Figure 8C) [205]. Collectively, these findings indicate that the design of novel BMBs should not focus solely on promoting osteoblastogenesis but rather adopt a holistic strategy that integrates both osteogenic and osteoclastic processes to achieve optimal outcomes.

### Angiogenesis

Studies have shown that vascular and bone regeneration are mutually complementary, as vascular networks provide nutrients and signals essential for new bone formation. Vascular endothelial growth factor (VEGF) is a key regulator of angiogenesis and also modulates essential bone regeneration and remodeling [206]. Notably, VEGF synergistically enhances BMP2-induced osteogenesis [207, 208]. Other molecules traditionally regarded as angiogenic regulators, such as SLIT3, have more recently been recognized for their roles in osteogenic signaling [209–211]. The reciprocal relationship can be further illustrated by cell membrane vesicles (CMVs): endothelial cell (EC)-derived CMVs carry the osteogenesis-promoting factor BMP2, while BMSC-derived CMVs deliver the angiogenic factor VEGF [212]. Together, these findings provide compelling evidence that osteogenesis and angiogenesis are tightly interconnected processes that cooperatively drive successful bone regeneration.

**Figure 9:**
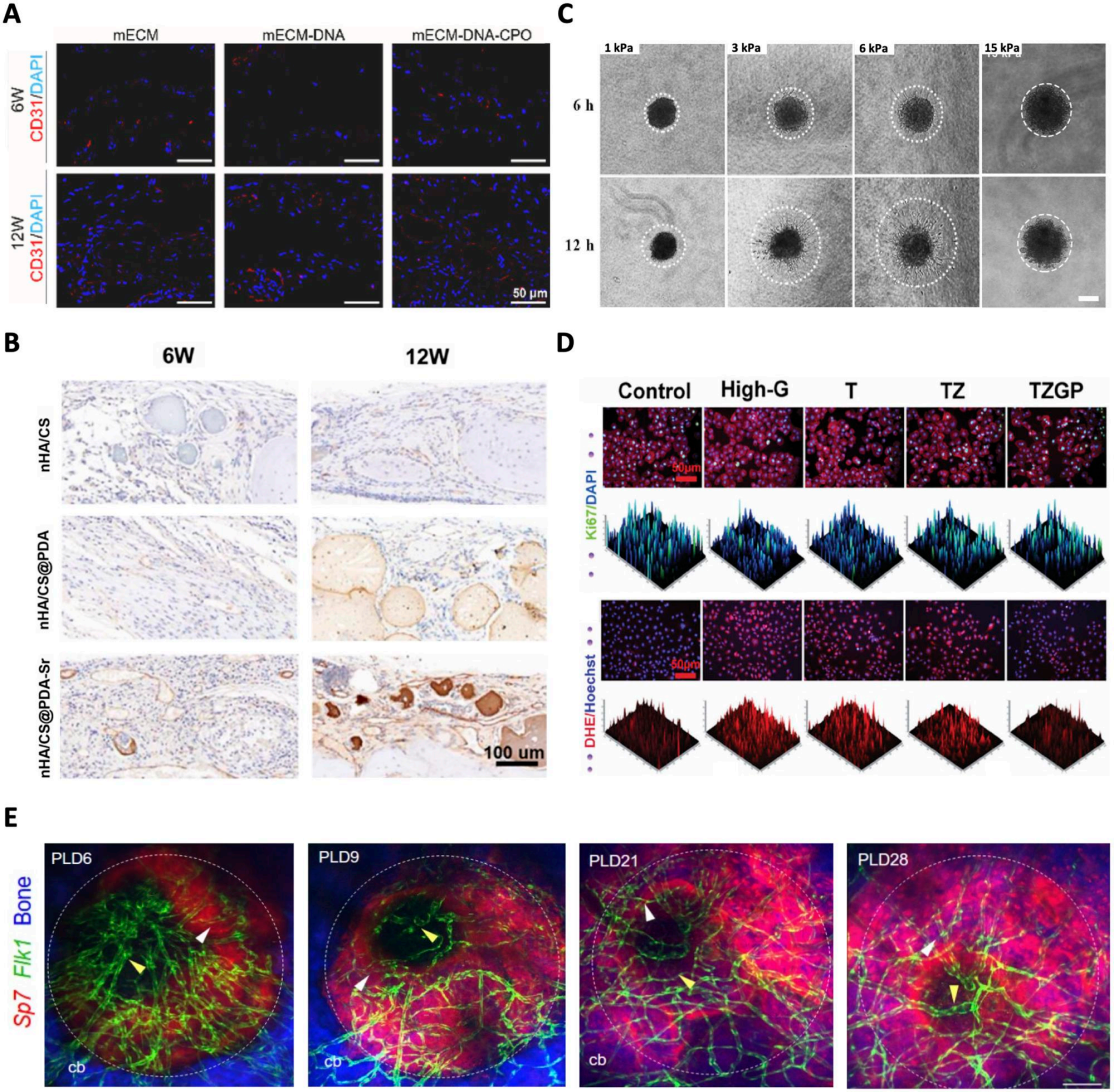
Applications and mechanisms of BMBs in angiogenesis. (**A**) CD31 immunofluorescence staining of a CPO-mECM hydrogel, demonstrating *in situ* vascularized calvarial defect repair; reproduced with permission from Ref. [216], Copyright 2025, Elsevier. (**B**) CD31 immunohistochemical staining showing that a strontium-containing mineralized scaffold promotes angiogenesis; reproduced with permission from Ref. [221], Copyright 2023, American Chemical Society. (**C**) Endothelial sprouting assay indicating that increased matrix stiffness enhances the invasive capacity of ECs. However, when stiffness reaches 15 kPa, both sprout number and invasion distance are reduced; reproduced with permission from Ref. [224], Copyright 2022, Springer Nature. (**D**) Immunofluorescence staining of Ki67 and DHE in human umbilical vein endothelial cells (HUVECs) cultured with or without α-TCP-mineralized gelatin scaffolds, representing cell proliferation and ROS level respectively. The presence of α-TCP scaffolds reduces intracellular ROS, thereby providing a favorable microenvironment for angiogenesis; reproduced with permission from Ref. [227], Copyright 2025, Wiley-VCH. (**E**) *In vivo* multiphoton microscopy revealing the progressive process of calvarial defect repair. At the early stage, Flk1-GFP^+^ vasculature invades and vascularizes the entire defect, whereas Sp7-mCherry^+^ osteoblasts and progenitor cells initially localize only at the bone edge; reproduced with permission from Ref. [228], Copyright 2024, Wiley-VCH.

Experiments have shown that metal ions commonly incorporated in BMBs (Ca^2+^, Zn^2+^, Mg^2+^, etc.) can promote paracrine between ECs and BMSCs through multiple signaling pathways, such as MAPK/ERK, PI3K/AKT, Wnt/β-catenin and JAK/STAT, that ultimately enhance osteoblast differentiation and vascular regeneration [213–215]. Gai *et al.* [216] mineralized decellularized extracellular matrix (mECM) with calcium phosphate oligomers (CPO), thus obtaining materials capable of promoting vascularized bone formation (Figure 9A). Mg^2+^ can also play a crucial role. For example, Yang *et al.* [217] loaded MgSiO_3_@Fe_3_O_4_ microspheres into mineralized PCL scaffolds to further improve angiogenesis. We have also confirmed the angiogenic function of Mg^2+^ with materials mineralized with amorphous calcium–magnesium phosphate [45]. Beyond these classical ions, Liu *et al.* [218] created BMBs with iron ion release, which could induce EC aggregation and upregulate HIF-1α that enhances VEGFA expression and promotes angiogenesis. Similarly, the presence of strontium ions (Sr^2+^) can act through the PI3K/AKT/mTOR and integrin β1/FAK/MAPK pathways to significantly enhance the angiogenic capacity of BMBs (Figure 9B) [219–221]. A strontium-apatite intrafibrillarly mineralized collagen/polycaprolactone methacrylate (PCLMA) Janus membrane we designed has also been shown to exhibit angiogenic effects both *in vitro* and *in vivo* [97]. These results firmly establish the central role of ion-mediated signaling in coordinating osteogenesis with angiogenesis and offer a rational strategy for designing next-generation BMBs.

The structural characteristics of BMBs and the microenvironment of defect sites are equally important. Yang *et al.* [222] fabricated linear and grid-patterned mineralized scaffolds and discovered that specific scaffold architecture mediated the angiogenic enhancement with Ca^2+^-dependent membrane channels. Since mineralization inevitably alters the mechanical properties of scaffolds, several studies have investigated how mechanical properties regulate angiogenesis. Lee *et al.* [223] demonstrated that stiffer matrices (2.47 kPa at 5% strain) facilitated the ingrowth of vascular sprouts. In contrast, Dzamukova *et al.* [224] reported that mechanical activation of PIEZO1 induced massive BMP1 release, which suppressed the VEGF pathway and drove the transition of blood vessels to limit vascularization. Similarly, Guo *et al.* [225] concluded that scaffold stiffness modulates vascular tip cell accumulation, with stiffness in the range of 1–6 kPa supporting angiogenesis, whereas stiffness at 15 kPa impedes it (Figure 9C). Beyond stiffness, Monaci *et al.* [226] summarized that hypoxic microenvironments influence multiple physiological processes, including angiogenesis, through hypoxia-inducible factors (HIFs), highlighting their importance in the design of BTE materials. Consistently, Wang *et al.* [227] fabricated α-TCP-based mineralized scaffolds that reduced intracellular reactive oxygen species (ROS) levels to facilitate angiogenesis (Figure 9D).

Moreover, numerous studies have been conducted to further elucidate the relationship between angiogenesis and bone regeneration. Gabriele Bixel *et al.* [228] employed multiphoton microscopy to track calvarial defect models in real time and found that angiogenesis consistently precedes bone healing. In fact, vascular formation was subsequently followed by the migration of osteoprogenitor cells and osteoblasts from the repair front into the vascularized defect area, revealing a temporal coupling between angiogenesis and osteogenesis (Figure 9E). Similarly, Schilling *et al.* [229] noted the spatiotemporal heterogeneity in the contributions of distinct vessel types during bone repair: CD31^+^EMCN^+^ vessels are the first to respond to injury and initiate angiogenesis but diminish once new bone mineralization occurs, while CD31^+^EMCN^-^ vessels emerge at later stages to support vessel branching and extension. These studies not only advance the current understanding of physiological bone healing but also lay the groundwork for optimizing the regulatory strategies of BMBs.

### Immunomodulation

**Figure 10:**
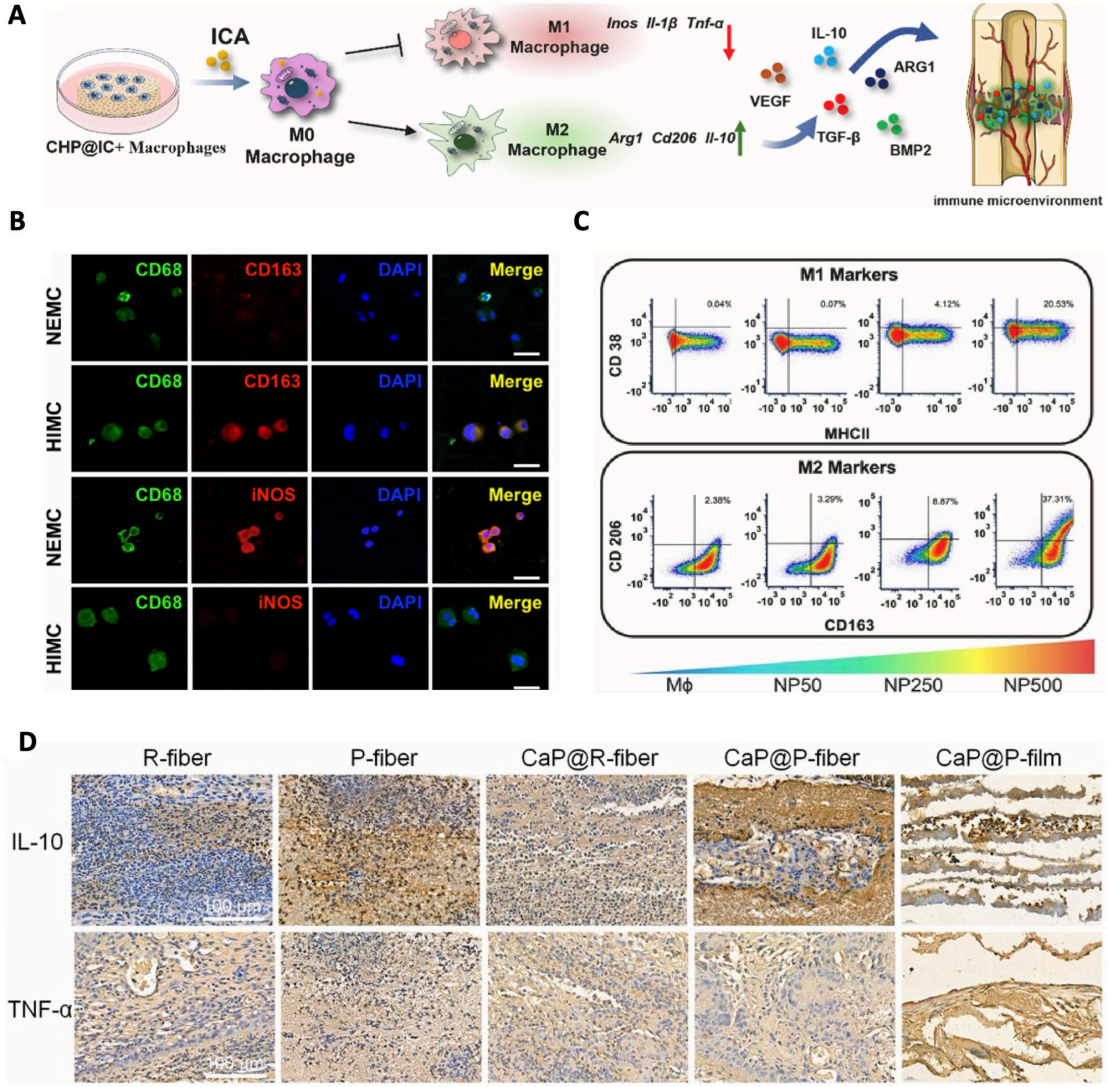
Immunomodulatory effects of BMBs. (**A**) Schematic illustration of how a HAp/collagen-based scaffold regulates the immune microenvironment, highlighting key cytokines involved in immunomodulation; reproduced with permission from Ref. [246], Copyright 2025, Elsevier. (**B**) Immunofluorescence staining of extrafibrillarly mineralized collagen (NEMC) and hierarchically intrafibrillarly mineralized collagen (HIMC). The HIMC group shows a higher prevalence of CD68^+^CD163^+^ M2 macrophages, whereas the NEMC group contains more CD68^+^iNOS^+^ M1 macrophages; reproduced with permission from Ref. [235], Copyright 2019, American Chemical Society. (**C**) Flow cytometric analysis of macrophages exposed to varying concentrations of amorphous calcium phosphate–chitosan nanoparticles (ACPC-NPs), demonstrating dose-dependent polarization toward both M1 and M2 phenotypes; reproduced with permission from Ref. [238], Copyright 2025, Elsevier. (**D**) IL-10, secreted mainly by M2 macrophages, and TNF-α, secreted mainly by M1 macrophages, reveal morphology-dependent immunomodulation; reproduced with permission from Ref. [247], Copyright 2022, Wiley-VCH.

In recent years, reports on BMBs modulating immune pathways to influence osteogenesis have been steadily increasing [230, 231]. The theoretical foundation of this immunoregulatory role lies in the distinct functions of macrophage subtypes: M1-type macrophages trigger intense inflammatory responses that can lead to bone destruction [232], whereas M2-type macrophages release anti-inflammatory factors and growth factors that promote osteogenesis and angiogenesis (Figure 10A) [233]. In previous studies, cobalt–β-TCP materials have been shown to enhance osteogenic differentiation of BMSCs; however, when macrophages were introduced into the evaluation system, the material adversely affected bone formation due to M1-type polarization [234]. This study further suggests that the immunomodulation of BMBs should be regarded as a critical indicator of *in vivo* osteogenic potential.

Jin *et al.* [235] found that intrafibrillarly mineralized materials with structural biomimetic features were more effective at activating M2-type macrophages than their extrafibrillarly mineralized counterparts, suggesting that both the minerals and their location have immunoregulatory effects (Figure 10B). Ca^2+^ can downregulate pro-inflammatory gene expression while upregulating anti-inflammatory factors, thereby promoting the macrophage phenotype transition from M1 to M2 at sites of injury [236]. Another study demonstrated that Ca^2+^ promotes BMSCs’ osteogenic differentiation via ERK-mediated macrophage activation [237]. Notably, the immunomodulatory effect of Ca^2+^ is concentration-dependent, intensifying as the ion level increases (Figure 10C and D) [238]. Other ions, such as Zn^2+^ and Sr^2+^, also influence macrophage activation and cytokine release, thereby participating in immunomodulation [239]. For example, Zn^2+^-coated polyetheretherketone induces M2 macrophage polarization by downregulating NF-κB expression or upregulating the JAK–STAT pathway [240], while Sr^2+^-doped submicron bioactive glass achieves a similar effect through NF-κB suppression [241]. Zhao *et al.* [242] further demonstrated that Sr^2+^–Zn^2+^ phosphate composites exhibit stronger M2 macrophage polarization than Sr^2+^– Ca^2+^ phosphate composites. Recent studies also indicate that Sr^2+^ and Zn^2+^ can promote osteogenesis independently of M2 macrophage polarization, whereas Ca^2+^ predominantly exerts its osteogenic effects through M2 macrophage-mediated pathways [243–245]. The findings highlight the distinct immunoregulatory mechanisms of different osteogenic ions. Given that these ions often serve as mineralization precursors or additives in BMBs, the materials inherently enhance osteogenesis through immunomodulation.

### Neuroregeneration

Numerous experimental studies have demonstrated that the presence of peripheral nerve fibers is critical for effective bone regeneration [248–250], with various neuropeptides and neural cells actively involved in this process [251]. Key biological factors include calcitonin gene-related peptide (CGRP) [252], substance P (SP) [253] and Schwann cells. For example, Wang *et al.* [254] reported that miRNAs and proteins in Schwann cell-derived exosomes can promote the osteogenic differentiation of BMSCs; Zhang *et al.* [255] showed that CGRP enhances the osteogenesis of BMSCs; and Li *et al.* [256] directly elucidated the close relationship between nerve repair and bone regeneration via the nerve growth factor (NGF)–Trk signaling pathway. Conversely, Fan *et al.* [257] demonstrated that miRNAs carried by BMSC-derived exosomes can create a favorable microenvironment for nerve repair through immunomodulation, thus suggesting the reciprocity of bone regeneration and neural repair. Recently, the regulatory role of the neuro–bone axis has drawn increasing attention and inspired the design of various BMBs.

**Figure 11:**
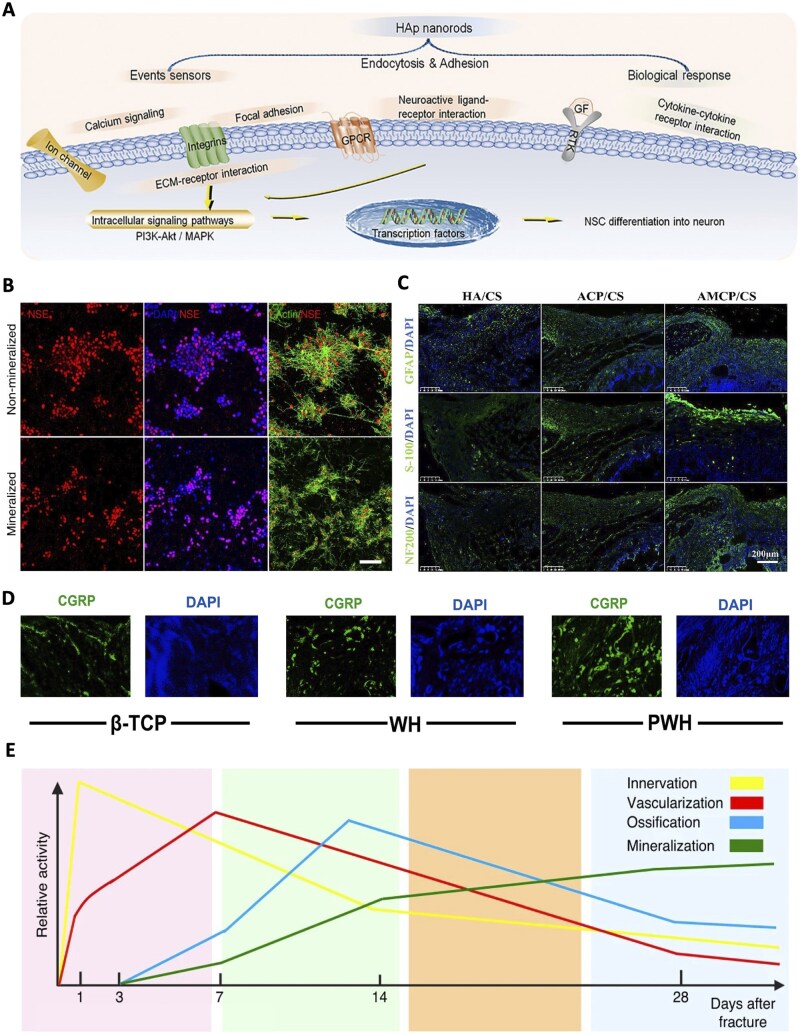
Functions and mechanisms of BMBs in neuroregeneration. (**A**) Schematic illustration showing how HAp nanorods enhance neuroregeneration of neural stem cells; reproduced with permission from Ref. [272], Copyright 2021, Wiley-VCH. (**B**) Representative immunofluorescence images of neuron-specific enolase (NSE) expression in nonmineralized and intrafibrillarly mineralized HAp–collagen scaffolds; reproduced with permission from Ref. [273], Copyright 2019, Springer Nature. (**C**) Immunofluorescence staining analysis demonstrating the effect of an AMCP/CS scaffold on neural factor expression. The release of Mg^2+^ ions enhances the neuroregenerative capacity of the scaffold; reproduced with permission from Ref. [258], Copyright 2024, American Chemical Society. (**D**) Immunofluorescence staining of CGRP showing that PWH promotes *in vivo* neuroregeneration; reproduced with permission from Ref. [265], Copyright 2023, Elsevier. (**E**) Line chart displaying the temporal sequence of biological events following a bone fracture; reproduced with permission from Ref. [270], Copyright 2023, Springer Nature.

In these biomaterials, mineral components are generally considered closely related to neural repair (Figure 11A and B). Yang *et al.* [258] fabricated amorphous magnesium–calcium pyrophosphate (AMCP)/cassava starch (CS) cellular scaffolds that not only exhibited osteogenic potential but also elevated neural repair marker NF200 and Schwann cell maturation marker S-100 (Figure 11C). Jing *et al.* [259] further demonstrated that GelMA–black phosphorus hydrogels enriched with Mg^2+^ promoted Schwann cell migration and NGF secretion, highlighting the role of Mg^2+^ in neuroregeneration. From a mechanistic perspective, Mg^2+^ has been shown to enhance CGRP release [252], which activates receptors on periosteum-derived stem cells and upregulates osteogenic differentiation genes such as RUNX2 [260]. This result established a molecular link between neural repair and bone regeneration. In addition to Mg^2+^, other neuroregulatory mediators are also engaged. Calmodulin-dependent protein kinase II (CaMKII) [261] and growth-associated protein 43 (GAP-43) [262] can mediate neuroprotection and neuroregeneration via NF-κB signaling and brain-derived neurotrophic factor (BDNF) respectively [263], while Ca^2+^ itself serves as a key messenger in neural stem cell differentiation and regeneration [264].

As for material design, Wang *et al.* [265] enhanced the neurogenic potential of whitlockite, which is capable of simultaneously releasing Ca^2+^, Mg^2+^ and phosphates, by introducing piezoelectricity (PWH) (Figure 11D). Building on these insights, Zhao *et al.* [266] developed a bilayer biomimetic scaffold, comprising a chitosan–HAp layer to replicate mineralized bone and an alginate–GelMA–whitlockite layer to mimic the periosteum with piezoelectricity. Beyond its pronounced capacity for bone regeneration, the scaffold demonstrates neuroregenerative effects that Mg^2+^ and Ca^2+^ jointly mediate via multiple signaling pathways. Other studies have established more refined *in vitro* loading models that stimulate piezoelectricity and have included a broader range of neurogenic differentiation–related markers, including Nestin, NEFL and Tubb3 [267, 268]. These approaches provide stronger evidence supporting the neuroregenerative potential of PWH. Building on the concept of the neuro–bone axis, Li *et al.* [269] also proposed to enhance neuroregeneration of BTE materials through the incorporation of bioactive ions, Schwann cell-derived exosomes, neurotrophic factors and relevant cell types. However, the function of BMBs depends not only on their composition but also on factors like surface topography, nanostructure and responsiveness to external stimuli, which are crucial for neural integration and regeneration. For instance, materials with mechanical properties below 500 Pa have been shown to promote neural stem cell differentiation into neurons, while porous materials with pore sizes around 300 nm facilitate axonal outgrowth [270]. Future efforts could, therefore, focus on engineering BMBs that systematically combine optimal mechanical cues, microscale and nanoscale topography and bioactive signaling to achieve coordinated neuro-osteogenic outcomes, potentially unlocking new opportunities for bone regeneration.

Recently, Li *et al.* [256] employed Thy1-YFP reporter mice to study the temporal relationship in bone healing. It has been concluded that neuroregeneration occurs before bone regeneration after injury (Figure 11E). After demonstrating the critical role of TrkA signaling in bone healing, the team’s recent work further identified neuron-derived fibroblast growth factor 9 (FGF9) as another key factor that directly links sensory nerves to bone healing [271]. As the molecular mechanisms underlying the close interplay between the neural system and bone have been elucidated, the identification and development of suitable BMBs are becoming a growing research focus.

Key biological processes in bone healing can be systematically regulated by BMBs through surface morphology, chemical composition and mechanical properties. The processes are dynamically coupled and temporally coordinated through intricate networks of cell–cell communication. Within this interplay, immunomodulation is an early dominant event to reshape the local microenvironment through macrophage polarization. For example, the establishment of an anti-inflammatory macrophage phenotype promotes osteogenic differentiation via IL-10, while simultaneously initiating angiogenesis through factors such as VEGF and platelet-derived growth factor BB (PDGF-BB) [274]. This transition marks the shift from immune regulation to tissue regeneration. In addition, EC-derived VEGF directly enhances the recruitment and differentiation of osteogenic cells [275], thereby establishing a functional coupling between angiogenesis and osteogenesis. Meanwhile, neuroregeneration contributes to vascular stabilization and bone regeneration by releasing factors including NGF [276]. Osteogenesis, in turn, exerts feedback regulation on immune cell behavior and vascular dynamics via bioactive factors such as BMP2 [277], which reinforce the stability and functionality of the regenerative microenvironment. Collectively, immunomodulation, angiogenesis, neuroregeneration and osteogenesis constitute a multidimensional regulatory network following BMB implantation and ultimately drive efficient bone healing.

## Clinical translation and restrictions of BMBs

The aging population, along with the rising occurrence of osteoporosis and complex bone defects, has increased the clinical need for effective, safe and easily accessible bone substitutes. As mentioned, although autografts and allografts generally yield better therapeutic results, their clinical use is limited by availability issues and concerns over disease transmission and graft rejection. These challenges highlight the need for engineered materials for bone regeneration. As a representative category in this field, BMBs exhibit favorable biocompatibility, tunable compositional and structural design and versatile functionality [278], which position them as promising candidates for translation from laboratory research to clinical application. We suggest that the translation of BMBs can be assessed based on the following aspects in clinical bone regeneration.

1. Biosafety: Biosafety should be regarded as a primary consideration in the translation of BMBs. This priority reflects not only the risk that toxic ingredients may directly cause tissue damage, but also the potential for biologically incompatible materials to trigger immune rejection and persistent inflammatory responses [279]. Such adverse effects disrupt cell behaviors and microenvironmental homeostasis, which are essential for bone regeneration, and ultimately compromise the therapeutic outcomes. Natural components, represented by collagen and various mineral ions, are intrinsic constituents of native bone and therefore generally exhibit reliable biosafety and favorable integration with host tissues. Some of them have also been proven biosafe in large-animal models and clinical trials [280, 281]. In contrast, the fabrication of synthetic polymers is often more complex and frequently involves multistep synthesis, chemical modification and the incorporation of exogenous agents such as crosslinkers, initiators or functional additives. These factors not only increase processing complexity but may also pose potential risks related to residual reagents, degradation byproducts or long-term accumulation. Therefore, their long-term biosafety and *in vivo* behavior still need systematic evaluation [282].
2. Standardized manufacturing: BMBs exhibit favorable reproducibility and strong potential for industrial-scale production. Firstly, their constituent materials are primarily sourced from well-established industrial products possessing consistent physicochemical properties and mature supply chains, thereby ensuring reliable batch-to-batch consistency during scale-up. Secondly, their fabrication processes are typically regulated by well-defined and quantifiable parameters. Critical variables, such as ion concentration, pH, temperature and reaction time, have been extensively documented to support process reproducibility. Thirdly, biomimetic mineralization enables precise regulation of nucleation and crystal growth, leading to the controlled assembly of mineral structures. Despite these advantages, the amorphous mineral precursors involved in the early stages are inherently unstable [283]. Their transient nature and sensitivity to local physicochemical environments may lead to variations in nucleation and crystallization, thereby introducing inconsistencies in mineralization outcomes.
3. Production efficiency: Enhancing production efficiency facilitates the manufacturing of high-yield biomaterials with superior properties at reduced cost and with simplified processing. This is closely linked to the commercialization potential of BMBs. Generally, BMBs are produced under physiological conditions, typically at moderate temperature and pressure. These mild conditions eliminate the need for high-temperature sintering or energy-intensive equipment, thereby reducing both energy consumption and capital investment compared with conventional industrial materials. In addition, biomimetic mineralization largely relies on spontaneous reactions in solution and requires limited external intervention. This feature contributes to a more streamlined processing workflow. However, production is constrained by the slow kinetics. In conventional SBF systems, apatite formation generally requires days to weeks, while even accelerated approaches still take hours to days. In addition, strict control over sterility, pH, temperature and ion concentrations is necessary throughout the process, which increases operational complexity and may ultimately limit the production efficiency and scalability of BMBs.
4. Customization: To be clinically applied, BMBs are increasingly expected to offer the capability for personalized customization. It allows them to adapt to diverse anatomical structures of defects and potentially broadens the indications for a material system. The Osteomimix platform designed by us has integrated CAD/CAM technology to perform precise cutting of BMBs to achieve morphological conformity with complex, irregular bone defects and provide a feasible route for clinical personalization [45]. With the advancement of additive manufacturing, an increasing number of BMBs can now be incrementally constructed via 3D or 4D printing [118]. This approach enhances processing efficiency while maintaining structural accuracy and enables the rapid fabrication of geometrically complex constructs. It should be noted, however, that the mechanical environment at different anatomical sites, the type of bone defect and patient-specific systemic factors—such as age, metabolic status and overall health—can all significantly influence the repair outcomes. Therefore, the selection and design of BMBs must still rely on comprehensive judgment from clinicians to achieve optimal therapeutic results [284].
5. Regulatory oversight: As bioactive materials continue to advance rapidly, regulatory frameworks are evolving accordingly, yet significant challenges remain. Variations in regulations across regions, coupled with the complex composition and rich bioactivity of BMBs, complicate both legislation and regulatory practice. For example, in China, passive orthopedic implants and related materials used for clinical bone regeneration are typically classified as Class III medical devices. These high-risk products are subject to stringent quality control and rigorous clinical evaluation requirements. Furthermore, many BMBs developed in laboratory settings not only serve structural roles but also exhibit multiple bioactive functions, such as promoting BMSC differentiation, enhancing EC proliferation and stimulating angiogenesis and neurogenesis. Such multifunctionality may lead to their classification as a combination of drug and medical device under regulatory frameworks. Compared to traditional biomaterials, BMBs necessitate a clear delineation of their passive properties and bioactive functions, accompanied by a comprehensive understanding of the underlying mechanisms [285]. Looking forward, a solid foundation can be established by promoting standardization and normalization of production processes and strengthening interdisciplinary collaboration between material scientists and regulatory experts. Such efforts will facilitate the safe clinical translation of BMBs and help realize their potential in regenerative medicine.

**Figure 12:**
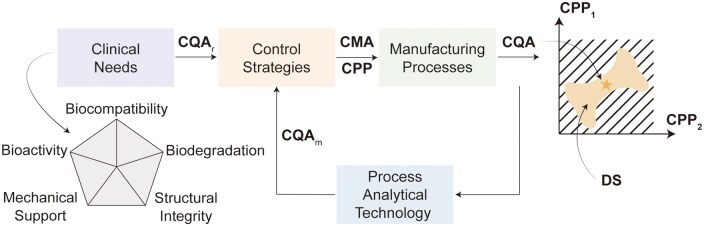
Schematic illustration of the QbD workflow to demonstrate the possible clinical transition of BMBs; reproduced with permission from Ref. [286], Copyright 2020, Springer Nature; reproduced with permission from Ref. [289], Copyright 2020, Wiley-VCH.

In laboratory settings, BTE materials are typically evaluated from five perspectives, namely biocompatibility, bioactivity, biodegradation, mechanical support and structural integrity [286]. These criteria reflect both their biological functions and their suitability for specific defect scenarios. As a representative subtype of BTE materials, BMBs are expected to satisfy the same considerations. In manufacturing and clinical applications, safety and efficacy are the two core requirements for medical products. Safety guarantees that patients are not exposed to unacceptable risks, while efficacy assesses the product’s ability to treat relevant clinical conditions in the target group [287]. Quality by Design (QbD) has been introduced into clinical translation as a systematic approach to guide both product design and manufacturing. Within this framework, clinical needs define the quality target product profile (QTPP). Critical quality attributes (CQAs), which are governed by critical manufacturing attributes (CMA) and critical process parameters (CPP), directly reflect the key characteristics of the product. Design space (DS), which represents the multidimensional range of material attributes and process parameters ensuring product quality, is defined through carefully planned experiments. Within this space, parameter variations are deemed acceptable and do not qualify as regulatory changes. The product quality also remains reliable. To further ensure consistency, process analytical technology is employed to monitor product quality in real time, thereby enabling robust control over manufacturing processes and ensuring consistent effectiveness [288–290]. The relationships among these concepts are summarized in Figure 12.

## Summary and outlook

Bone is a vital organ that provides essential mechanical support and physiological functions, yet the repair of bone defects remains a major clinical challenge due to the limitations of current therapeutic strategies. Driven by the demand for safer and more effective solutions, BMBs have emerged as a promising paradigm grounded in the intricate biological blueprint of bone. Particular attention is given to how these materials regulate key biological processes that collectively shape the regenerative microenvironment. In this context, bone healing is increasingly recognized not as a single biological event but as a coordinated and dynamic interplay among multiple biological systems. It underscores the importance of designing multifunctional biomimetic platforms.

This review begins with an overview of the biological foundations of bone and biomineralization, with emphasis on recent mechanism insights. Biomimetic mineralization strategies are then systematically summarized, including both conventional approaches and emerging techniques, with a critical comparison of their respective advantages and limitations. Subsequently, BMBs are categorized into biopolymers, bioactive ceramics and metals. This classification enables a structured comparison of their structural characteristics and functional performance. The discussion then highlights how these materials actively regulate osteogenesis, angiogenesis, immunomodulation and neuroregeneration. These events collectively shape the regenerative microenvironment in a coordinated manner. The intrinsic interdependence suggests that the design of BMBs should shift from optimizing isolated processes to orchestrating multiple biological events in a coordinated sequence, thus facilitating comprehensive bioactivity validation and improving preclinical assessment.

Despite significant advances, the clinical translation of BMBs remains constrained by a series of interrelated challenges of biological design, materials engineering and regulatory considerations. Most current BMBs fail to recapitulate the spatiotemporal sequence of natural bone healing or adapt to diverse pathological microenvironments. In addition, their predominantly static structures limit responsiveness to dynamic *in vivo* conditions, while the increasing structural and functional complexity of these materials poses substantial difficulties for standard and scalable manufacturing. Coupled with insufficient long-term safety data and the lack of well-defined regulatory frameworks, these factors collectively hinder the transition of BMBs from laboratory research to reliable clinical applications.

Looking ahead, several key directions could guide next-stage development. Improving biomimicry across nanoscale, microscale and macroscale hierarchies remains crucial for more accurately replicating native bone structure. Meanwhile, future materials are expected to shift from passive scaffolds to actively responsive systems capable of adapting to or even interacting with the changing biophysical and biochemical environment. Expanding functional capabilities—by incorporating piezoelectric, photo-responsive or thermoelectric properties—may further increase therapeutic potential. Overcoming these challenges will require more than just incremental progress. It calls for mechanism-guided material design, integrated manufacturing strategies and a move toward multidisciplinary evaluation models that reflect the full complexity of bone healing. Ultimately, success will depend not only on innovations in multi-dimensional material design but also on a deeper, more systematic understanding of biomineralization itself.

## Supplementary Material

rbag093_Supplementary_Data
