# Supplementary material for: Advances in biomimetic mineralized biomaterials for bone tissue engineering
Source: Regen Biomater. 2026 May 12;13:rbag093. doi: 10.1093/rb/rbag093 (PMC13275137; doi:10.1093/rb/rbag093)
Supplement: rbag093_Supplementary_Data [file rbag093_supplementary_data.docx]

**Advances in Biomimetic Mineralized Biomaterials for Bone Tissue Engineering**

*Zefang Li^a,1^, Wei Sun^a,1^, Shuaicong Huang^a,1^, Yaning Zhao^a^, Xiaoyi Wu^a^, Cui Huang^a,*^, Hongye Yang^a,*^*

^a^ State Key Laboratory of Oral & Maxillofacial Reconstruction and Regeneration, Key Laboratory of Oral Biomedicine Ministry of Education, Hubei Key Laboratory of Stomatology, School & Hospital of Stomatology, Wuhan University, Wuhan, 430079, China*.*

^1^ These authors contributed equally to this review.

**Supplementary Information**

| **Supplementary Table 1** Overview of the reported fabrication strategies and functional properties of representative BMBs | | | | | | | | |
| --- | --- | --- | --- | --- | --- | --- | --- | --- |
| **Base Material** | **Composite Material** | **Fabrication Strategy** | **Physical & Mechanical Properties** | **Biological Properties** | | | | **Ref** |
|  |  |  |  | **Osteogenesis** | **Angiogenesis** | **Immunomodulation** | **Neuroregeneration** |  |
| Collagen | Mineralized tendon with ACP | Freeze-thaw decellularization;  PILP mineralization | Elastic modulus:  (0.05–0.1) GPa  Hardness:  (0.02–0.03) GPa | BV/TV: ~40% Tb.Th: ~0.04 cm Tb.Sp: ~0.005 cm | - | - | - | [1] |
|  | Intrafibrillarly strontium-apatite-mineralized collagen/PCLMA Janus membrane | Chemical crosslinking;  PILP mineralization | Elastic modulus:  25.63 MPa  Tensile strength:  1.54 MPa | BV/TV: 72.19%  BMD: 388.62 mg/cm³ | Nodes: ~400 Junctions: ~110  Meshes: ~40 Branching length of vessels: ＞6000 μm | Upgrade: CD206, Arg-1, IL-10, TGF-β1, VEGFA, PDGFBB  Downgrade: CD80, CD11c, IL-1β, iNOS, TNFα | - | [2] |
|  | Multiphase mineralized collagen scaffold with copper sulfide nanoparticle | Self-assembly;  PILP mineralization | Pore size:  (124.16±7.67) μm  Young's modulus:  (7.78–12.09) GPa | BV/TV: (87.17±8.60)% | Upgrade: IL-10, CD206  Downgrade: IL-1β, TNF-α | - | - | [3] |
| Gelatin | HAp/nanofibrous gelatin scaffold | Thermally induced phase separation;  SBF soaking | Pore size:  250–420 μm  Compressive modulus:  ~1400 kPa | Upgrade: BSP, OCN | - | - | - | [4] |
|  | Mineralized methacrylated alginate/GelMA hydrogel | DLP printing;  PILP mineralization & Enzyme-induced mineralization | Compressive modulus:  (3–4) MPa | BV/TV: 51.15% | - | - | - | [5] |
|  | Magnesium-modified black phosphorus/GelMA hydrogel | UV irradiation;  SBF soaking | Pore size:  (250–300) μm  Elastic modulus:  (1.5–2) kPa | BV/TV: ~35% | - | - | Upgrade: NGF, BDNF, S100, CGRP^+^ nerve fibers | [6] |
| Silk fibroin | Post-mineralized sodium alginate/silk fibroin scaffold | 3D printing;  Wet chemical method | Compressive modulus:  (750–1000) kPa | Upgrade: Runx2, OPN, OCN, OSX, Col1a | - | - | - | [7] |
|  | HAp/regenerative silk fibroin hydrogel | Chemical crosslinking;  SBF soaking | Compressive strength:  ~4.5 MPa | BV/TV: ~40%  BMD: ~250 mg/cm³ | - | - | - | [8] |
|  | ACP/platelet-rich plasma/oxidized silk fibroin hydrogel | Physical crosslinking;  PILP mineralization | Pore size:  (181.17±83.74) nm  Storage modulus:  ~7.6 kPa | BV/TV: 46.01%  BMD: 420.32 HA/cm³ | Meshes: 44.3  Nodes: ~700 | - | - | [9] |
| Cellulose | Calcium/zinc hybrid HAp/oxidized bacterial cellulose scaffold | Ion crosslinking;  SBF soaking | - | BV/TV: 52.5% | - | Upgrade: CD206, Arg-1, TGF-β  Downgrade: CD80, iNOS, IL-1β | - | [10] |
|  | HAp/macroporous bacterial cellulose scaffold | Freeze-drying;  SBF soaking | Pore size:  (100–300) μm  Compressive modulus:  (217.4±52.7) kPa  Tensile stress:  ~30 MPa  Elastic modulus:  ~35 MPa | BV/TV: ~50% BMD: 564.4 mg/cm³ | - | - | - | [11] |
|  | Anisotropic HAp/white wood hydrogel | Chemical crosslinking;  Wet chemical method | **Along fibers**:  Tensile strength:  (67.8±1.0) MPa  Elastic modulus:  (670±11) MPa Compressive strength:  (39.5±0.9) MPa  **Perpendicular to fibers**:  Tensile strength:  (13.2±1.2) MPa Elastic modulus:  (7.4±1.2) MPa  Compressive strength:  (27.8±0.8) MPa | BV/TV: ~41.3%  BMD: ＞350 mg/cm³ | - | - | - | [12] |
| Chitin | HAp/nanofibrous chitin microsphere | Thermally induced self-assembly;  Wet chemical method | Diameter:  70 μm  Pore size:  (10–20) μm | *In vivo* osteogenesis in rabbit radius defect model for 12 weeks | - | - | - | [13] |
|  | Amorphous calcium carbonate/chitin crab cuticle-derived membrane | Demineralization | Tensile strength:  23.4 MPa  Interfacial adhesion strength:  ~500 kPa | BV/TV: 72.82% | - | - | - | [14] |
|  | Mineralized chitin nanocrystal/chitosan scaffold | Chemical crosslinking;  Wet chemical method | **Rod-like α-chitin**:  Compressive modulus:  (191±10) kPa  **Sphere-like β-chitin**:  Compressive modulus:  (182±8) kPa | *In vitro* osteogenic differentiation of human adipose-derived mesenchymal stem cells for 7, 14 and 21 days | - | - | - | [15] |
| Chitosan | HAp/chitosan scaffold | Free-drying;  Enzyme-induced mineralization | - | BV/TV: (54.32 ± 3.41)% | - | - | - | [16] |
|  | HAp/chitosan nanocomposite scaffold | Freeze-drying;  Wet chemical method | - | *In vitro* ALP and OCN expression in BMSCs | - | Upgrade: IL-4, IL-10  Downgrade: TGF-β | - | [17] |
|  | ACP/carboxymethyl chitosan hydrogel with BMP9 | pH-induced self-assembly;  PILP mineralization | Storage modulus:  ~10 kPa | Mean bone density: ＞1000 HU  Trabecular bone area: (1.0~1.2)×10^6^ a.u. | - | - | - | [18] |
| Synthetic Polymer | Fluffy HAp/PLGA scaffold | Electrospinning;  SBF soaking | Pore size:  (70±20) μm  Compressive modulus:  830 Pa | *In vivo* osteogenesis in rabbit tibia defect model for 12 weeks | - | - | - | [19] |
|  | ACP/poly (octomethylene citrate) scaffold | Spontaneous mineralization | Pore size:  266.44 μm  Compressive strength:  17.05 MPa  Compressive modulus:  703.31 MPa | BV/TV: (29.03±3.95)%  Tb. Th: (0.19±0.02) mm | Junction: ~70  Branching length of vessels: ＞3000 μm | - | - | [20] |
|  | CaP/PLA scaffold with BMP2 | 3D printing;  SBF soaking | Pore size:  ~500 μm  Compressive modulus:  (0.510±0.11) GPa  Compressive strength:  (18.22±2.67) MPa | BV/TV: (44.85±11.09)% | - | - | - | [21] |
|  | HAp/polydopamine/PLLA scaffold | Electrospinning;  Piezoelectrically-induced mineralization | Young’s modulus:  5.4 GPa  Output voltage:  190 mV | BV/TV: (94.4±3.1)% | Upgrade: VEGF | Upgrade: CD206  Downgrade: CD86 | - | [22] |
| **Abbreviations**: BV/TV, bone volume/total volume; Tb.Th, trabecular thickness; Tb.Sp, trabecular separation; BMD, bone mineral density; BSP, bone sialoprotein; OCN, osteocalcin; OPN, osteopontin, also known as secreted phosphoprotein 1 (SPP1); OSX, osterix; Runx2, runt-related transcription factor 2; CD11c, cluster of differentiation 11c, also known as integrin αX (ITGAX); CD80, cluster of differentiation 80, also known as B7-1; CD86, cluster of differentiation 86, also known as B7-2; CD206, cluster of differentiation 206, also known as mannose receptor C-type 1 (MRC1); Arg-1, arginase-1; iNOS, inducible nitric oxide synthase, also known as nitric oxide synthase 2 (NOS2); IL-1β, interleukin-1 beta; IL-4, interleukin-4; IL-10, interleukin-10; TGF-β1, transforming growth factor beta 1; VEGF, vascular endothelial growth factor; VEGFA, vascular endothelial growth factor A; PDGF-BB, platelet-derived growth factor BB; TNF-α, tumor necrosis factor alpha; NGF, nerve growth factor; BDNF, brain-derived neurotrophic factor. | | | | | | | | |

References

1. Chen Y, Zhang Y, Chen X, Huang J, Zhou B, Zhang T, Yin W, Fang C, Yin Z, Pan H, Li X, Shen W, Chen X. Biomimetic intrafibrillar mineralization of native tendon for soft-hard interface integration by infiltration of amorphous calcium phosphate precursors. *Adv Sci* **2023**;10:e2304216.

2. Zhao Y, Sun W, Wu X, Gao X, Song F, Duan B, Lu A, Yang H, Huang C. Janus membrane with intrafibrillarly strontium-apatite-mineralized collagen for guided bone regeneration. *ACS Nano* **2024**;18:7204-22.

3. Wang Q, Li Z, Zhang Y, Guo H, Chen L, Zhang H, Liu H, Li C, Zhang S, Shi X, Feng L, Qi M, Luo D, Di P, Liu Y. Multiphase mineralized collagen scaffold for infected bone regeneration through oxidative metabolism modulation. *Adv Funct Mater* **2025**;35:2500593.

4. Liu X, Smith LA, Hu J, Ma PX. Biomimetic nanofibrous gelatin/apatite composite scaffolds for bone tissue engineering. *Biomaterials* **2009**;30:2252-8.

5. Wang L, Li D, Huang Y, Mao R, Zhang B, Luo F, Gu P, Song P, Ge X, Lu J, Yang X, Fan Y, Zhang X, Wang K. Bionic mineralized 3D‐printed scaffolds with enhanced in situ mineralization for cranial bone regeneration. *Adv Funct Mater* **2023**;34:2309042.

6. Jing X, Xu C, Su W, Ding Q, Ye B, Su Y, Yu K, Zeng L, Yang X, Qu Y, Chen K, Sun T, Luo Z, Guo X. Photosensitive and conductive hydrogel induced innerved bone regeneration for infected bone defect repair. *Adv Healthc Mater* **2023**;12:e2201349.

7. Shi R, Cai X, He G, Guan J, Liu Y, Lu H, Mao Z, Li Y, Guo H, Hai Y. Extrusion printed silk fibroin scaffolds with post-mineralized calcium phosphate as a bone structural material. *Int J Bioprint* **2022**;8:596.

8. Chen W, Li J, Meng D, Liu F, Huang L, Wang N, Mo G, Liang H, Su D, Zhang J, Jiang L. Biomineralized silk fibroin hydrogel with ultra-high strength for supporting bodyweight-bearing bone defects. *Int J Biol Macromol* **2025**;330:147729.

9. Zhu Y, Gu H, Yang J, Li A, Hou L, Zhou M, Jiang X. An injectable silk-based hydrogel as a novel biomineralization seedbed for critical-sized bone defect regeneration. *Bioact Mater* **2024**;35:274-90.

10. Luo C, Li YM, Jiang K, Wang K, Kuzmanovic M, You XH, Zhang Y, Lei J, Huang SS, Xu JZ. ECM-inspired calcium/zinc laden cellulose scaffold for enhanced bone regeneration. *Carbohydr Polym* **2024**;331:121823.

11. Xun X, Li Y, Ni M, Xu Y, Li J, Zhang D, Chen G, Ao H, Luo H, Wan Y, Yu T. Calcium crosslinked macroporous bacterial cellulose scaffolds with enhanced in situ mineralization and osteoinductivity for cranial bone regeneration. *Compos Part B Eng* **2024**;275:111277.

12. Wang X, Fang J, Zhu W, Zhong C, Ye D, Zhu M, Lu X, Zhao Y, Ren F. Bioinspired highly anisotropic, ultrastrong and stiff, and osteoconductive mineralized wood hydrogel composites for bone repair. *Adv Funct Mater* **2021**;31:2010068.

13. Duan B, Shou K, Su X, Niu Y, Zheng G, Huang Y, Yu A, Zhang Y, Xia H, Zhang L. Hierarchical microspheres constructed from chitin nanofibers penetrated hydroxyapatite crystals for bone regeneration. *Biomacromolecules* **2017**;18:2080-9.

14. Wang S, Yan K, Liu X, Chen X, Wu X, Zhao Y, Zhao X, Duan B, Zhou J, Yang H. Crab cuticle‐derived bilayer membrane orchestrates spatiotemporal maintenance and osteogenic activity for guided bone regeneration. *Adv Funct Mater* **2025**;36:e23211.

15. Olza S, Hadj Bouzidi NM, Rubatat L, Pellerin V, Montejo U, Alonso-Varona A, Fernandes SCM. Mineralized chitin nanocrystals enhance osteoinductive ability of chitosan 3D porous biohybrid scaffolds for bone tissue regeneration. *Carbohydr Polym* **2025**;366:123911.

16. Li N, Zhou L, Xie W, Zeng D, Cai D, Wang H, Zhou C, Wang J, Li L. Alkaline phosphatase enzyme-induced biomineralization of chitosan scaffolds with enhanced osteogenesis for bone tissue engineering. *Chem Eng J* **2019**;371:618-30.

17. Soriente A, Fasolino I, Gomez-Sanchez A, Prokhorov E, Buonocore GG, Luna-Barcenas G, Ambrosio L, Raucci MG. Chitosan/hydroxyapatite nanocomposite scaffolds to modulate osteogenic and inflammatory response. *J Biomed Mater Res A* **2022**;110:266-72.

18. Zhao C, Qazvini NT, Sadati M, Zeng Z, Huang S, De La Lastra AL, Zhang L, Feng Y, Liu W, Huang B, Zhang B, Dai Z, Shen Y, Wang X, Luo W, Liu B, Lei Y, Ye Z, Zhao L, Cao D, Yang L, Chen X, Athiviraham A, Lee MJ, Wolf JM, Reid RR, Tirrell M, Huang W, de Pablo JJ, He TC. A pH-triggered, self-assembled, and bioprintable hybrid hydrogel scaffold for mesenchymal stem cell based bone tissue engineering. *ACS Appl Mater Interfaces* **2019**;11:8749-62.

19. Tao Y, Jia M, Shao-Qiang Y, Lai CT, Hong Q, Xin Y, Hui J, Qing-Gang C, Jian-Da X, Ni-Rong B. A novel fluffy PLGA/HA composite scaffold for bone defect repair. *J Mater Sci Mater Med* **2024**;35:16.

20. Chen J, Xian G, Xiao Z, Ge F, Yuan S, Li B, Liang X, Cai Z, Zhang N, Zhang L, Li ZA, Deng L, Zeng C, Xie D. Biomineralization-inspired scaffolds using citrate-based polymers to stabilize amorphous calcium phosphate promote osteogenesis and angiogenesis for bone defect repair. *Bioact Mater* **2026**;56:260-76.

21. Maia-Pinto MOC, Brochado ACB, Teixeira BN, Sartoretto SC, Uzeda MJ, Alves A, Alves GG, Calasans-Maia MD, Thire R. Biomimetic mineralization on 3D printed PLA scaffolds: on the response of human primary osteoblasts spheroids and in vivo implantation. *Polymers* **2020**;13:74.

22. Cui X, Xu L, Shan Y, Li J, Ji J, Wang E, Zhang B, Wen X, Bai Y, Luo D, Chen C, Li Z. Piezocatalytically-induced controllable mineralization scaffold with bone-like microenvironment to achieve endogenous bone regeneration. *Sci Bull* **2024**;69:1895-908.
